# Supplementary material for: Turning copper into an efficient and stable CO evolution catalyst beyond noble metals
Source: Nat Commun. 2024 Jul 17;15:5998. doi: 10.1038/s41467-024-50436-4 (PMC11252372; doi:10.1038/s41467-024-50436-4)
Supplement: Supplementary file 1 — Supplementary Information [file 41467_2024_50436_MOESM1_ESM.pdf]

Supplementary Materials for

Turning copper into an efficient and stable CO evolution catalyst beyond noble metals

Jing Xue[1,2†], Xue Dong[3,†], Chunxiao Liu[1], Jiawei Li[1], Yizhou Dai[1], Weiqing Xue[1], Laihao Luo[1], Yuan Ji[1], Xiao Zhang[4], Xu Li[1], Qiu Jiang[1], Tingting Zheng[1], Jianping Xiao[3,5]*, Chuan Xia[1,*]

*Corresponding author: E-mail: chuan.xia@uestc.edu.cn (C.X.); xiao@dicp.ac.cn (J.X.)
†These authors contributed equally to this work.

Supplementary Note

As reported in previous literature[1], well-mixed multi-component alloys have relatively shallow negative ΔH values among the constituting elements, which prevents phase separation. Moreover, due to the high entropy of these alloys, the entropy contribution (TΔS) can be significant. Therefore, in our case, entropy plays a more dominant role in phase stability than does ΔH.

We also provide additional details on the mixing entropy (ΔS_{mix}) of different catalysts in this work. For comparison, we calculated ΔS_{mix} using the following equation[2]:

$$\Delta S_{mix} = -R \cdot \Sigma X_i \cdot \ln(X_i)$$

where R is the molar gas constant and X_i represents the mole ratio of the single-atom components in the alloy system. Given the above, the as-calculated ΔS_{mix} values of the three samples are 0.1347 R ($Cu_{97}Pd_3$), 0.1985 R ($Cu_{95}Sb_5$), and 0.3317 R ($Cu_{92}Sb_5Pd_3$). The mixing entropy increases as the number or ratio of single-atom components increases.

Using the formula for free energy (ΔG = ΔH - TΔS), we can infer that when ΔH is shallow and similar among the three SAA catalysts, the ΔG of the alloy system decreases as ΔS_{mix} increases with additional single-atom additions.

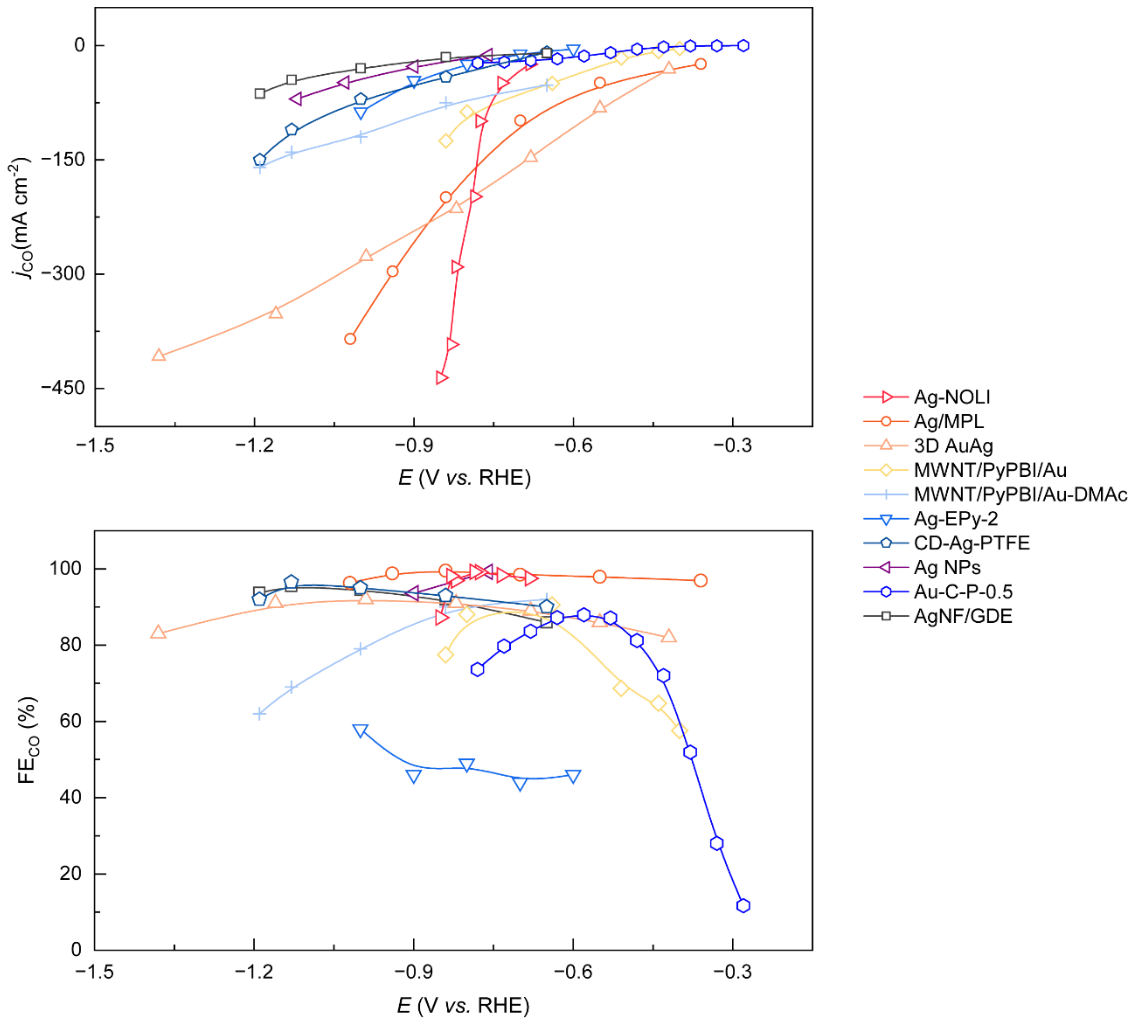

Supplementary Fig. 1 | Electrocatalytic performance of noble metal catalysts in the CO_2RR to CO in flow cell systems. The following reference catalysts were used: Ag-NOLI (1 M $KHCO_3$)[3], Ag/MPL (0.1 M $KHCO_3$)[4], 3D AuAg (1 M $KHCO_3$)[5], MWNT/PyPBI/Au (2 M $KHCO_3$)[6], MWNT/PyPBI/Au-DMAc (1 M KCl)[7], Ag-EPy-2 (0.1 M $KHCO_3$)[8], CD-Ag-PTFE (1 M $KHCO_3$)[9], Ag NPs (2 M $KHCO_3$)[10], Au-C-P-0.5 (1 M $KHCO_3$)[11] and AgNF/GDE (1 M KCl)[12]. As shown, noble metals tend to be inactive and susceptible to the hydrogen evolution reaction at high production rates, which compromises the CO selectivity.

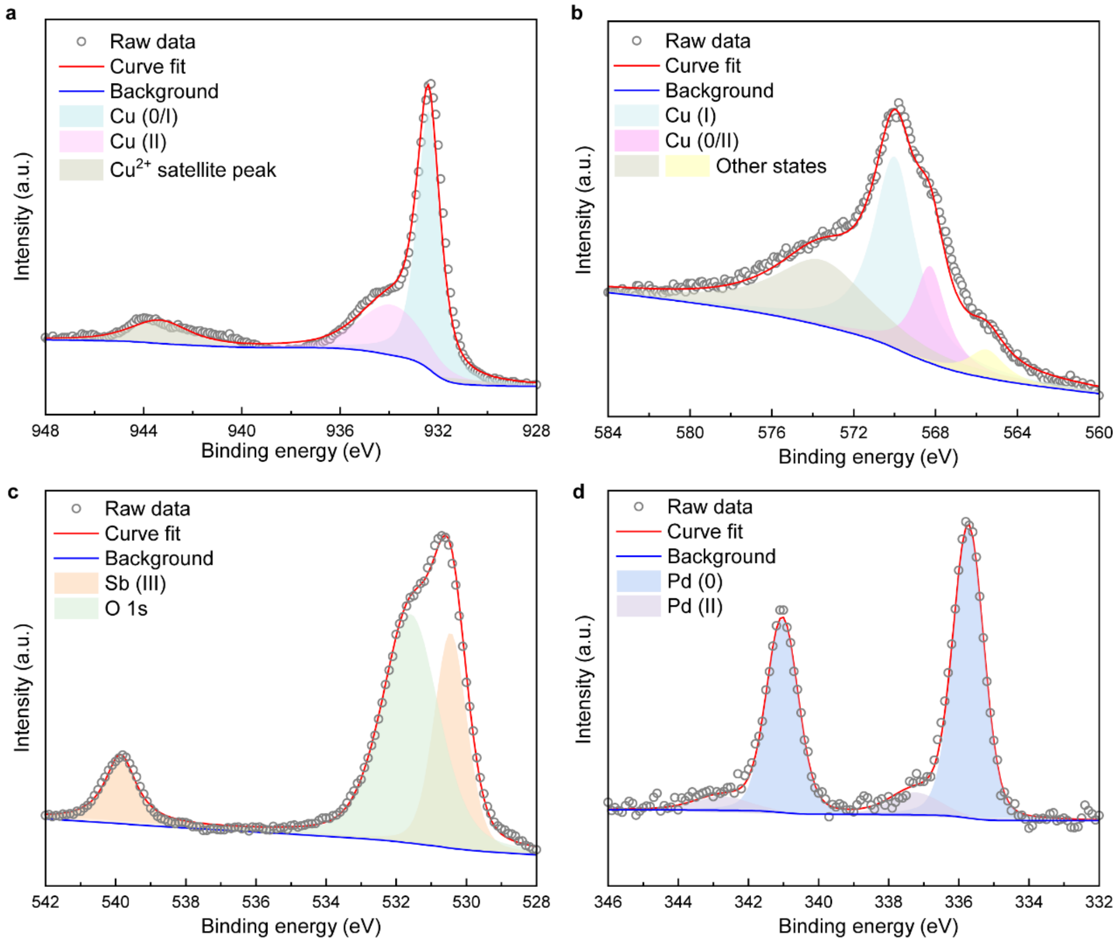

Supplementary Fig. 2 | X-ray photoelectron spectroscopy results for Cu 2*p* (a), Cu Auger (b), Sb 3*d* (c) and Pd 3*d* (d) on the $Cu_{92}Sb_5Pd_3$ catalyst. These results together demonstrate the successful introduction of two metal components, Pd and Sb, into this trimetallic catalyst. Note that a certain degree of unavoidable oxidation on the sample surface was observed due to the oxygen susceptibility of the Cu nanocrystal surface once exposed to air.

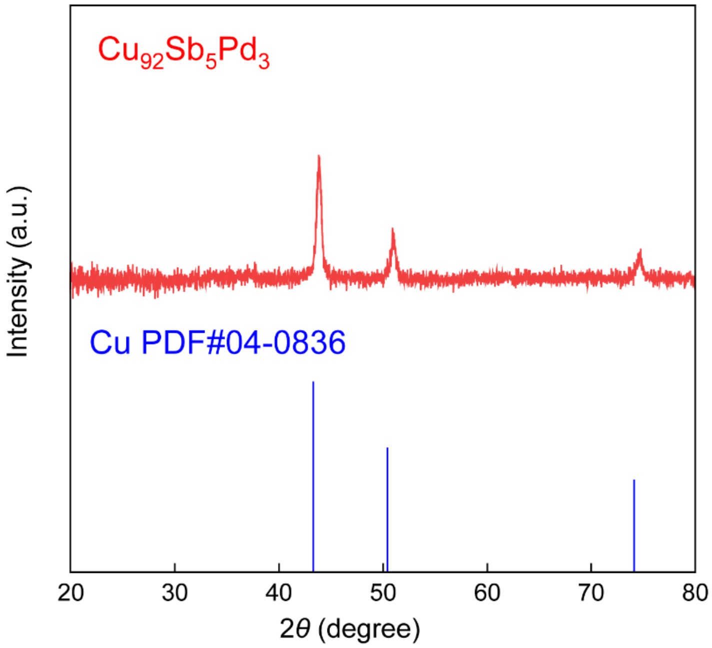

Supplementary Fig. 3 | XRD pattern of the as-synthesized $Cu_{92}Sb_5Pd_3$ catalyst. Only a Cu crystal structure (PDF 04-0836) appeared, which excluded the formation of either Sb or Pd nanoparticles and verified that the bulk phase alloy remained unoxidized.

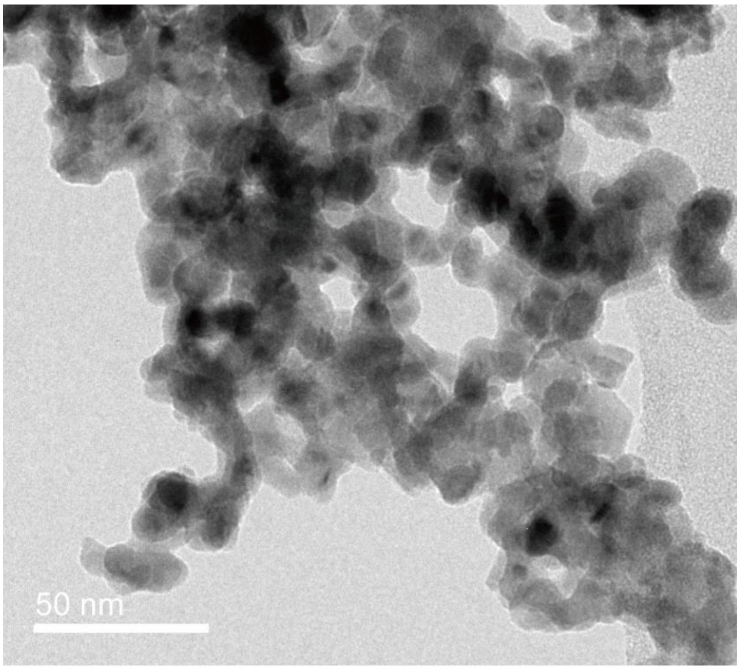

Supplementary Fig. 4 | TEM images of the as-synthesized $Cu_{92}Sb_5Pd_3$ catalyst. The sizes ranged from 10 to 20 nm.

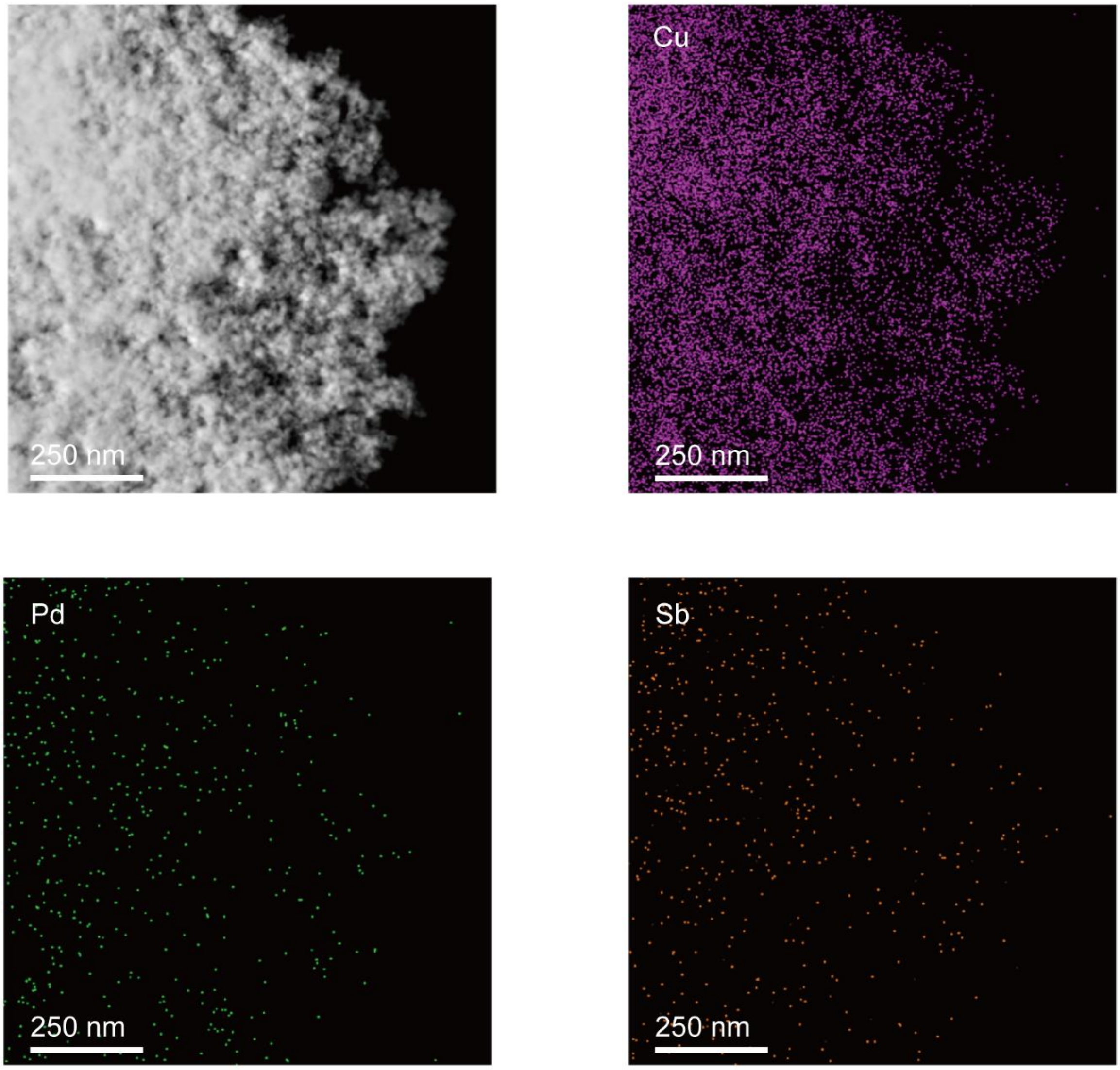

Supplementary Fig. 5 | Large-scale EDS mapping of the as-synthesized $Cu_{92}Sb_5Pd_3$ catalyst. No segregation was detected, precluding the presence of Sb or Pd particles.

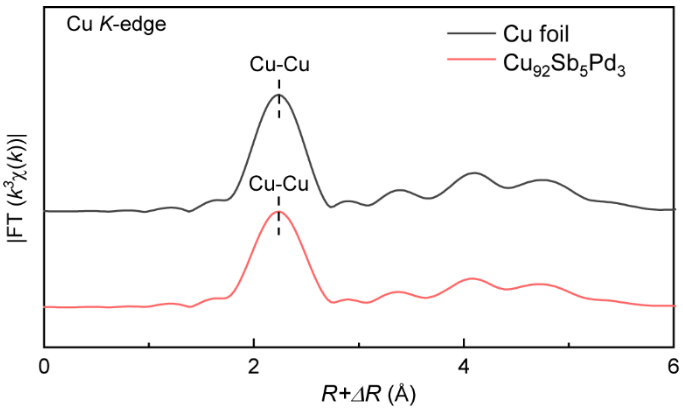

Supplementary Fig. 6 | Cu *K*-edge EXAFS spectra of the $Cu_{92}Sb_5Pd_3$ catalyst and Cu foil for comparison. Only the sole existence of Cu-Cu bonds (~2.24 Å) was shown, without any observation of copper oxides.

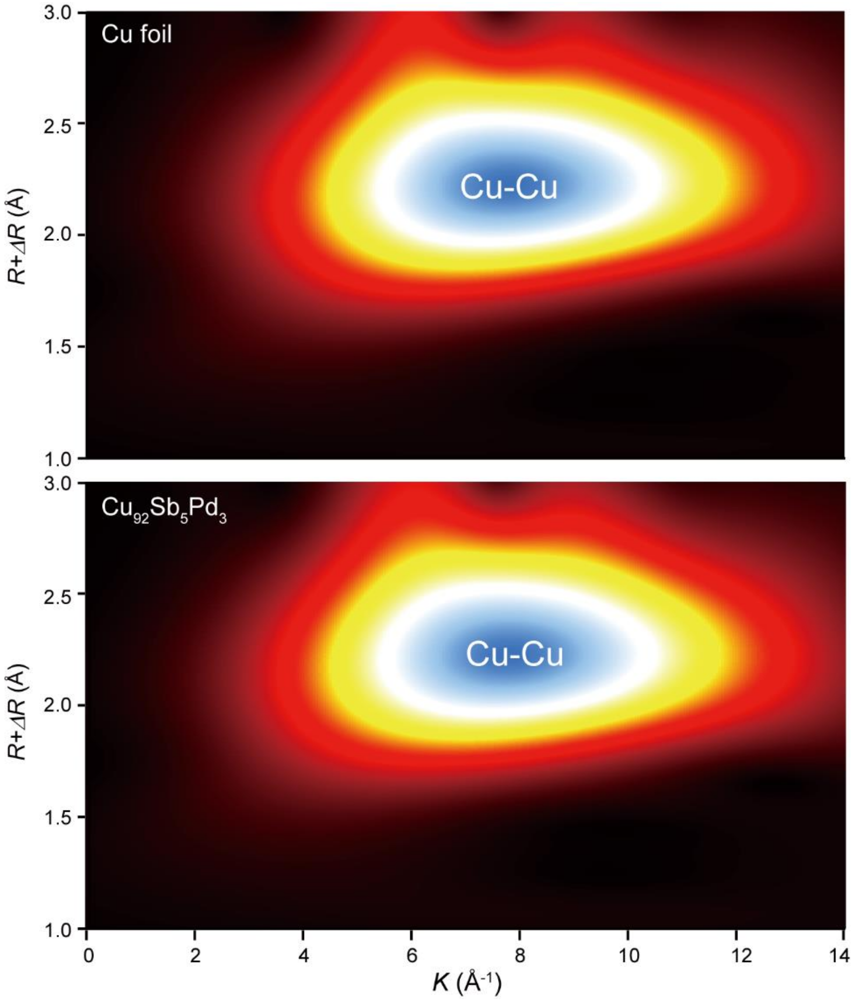

Supplementary Fig. 7 | EXAFS WT results of the $Cu_{92}Sb_5Pd_3$ catalyst and Cu foil for comparison. The two profiles are considerably similar. As above, only Cu-Cu bonds without any Cu-O were found.

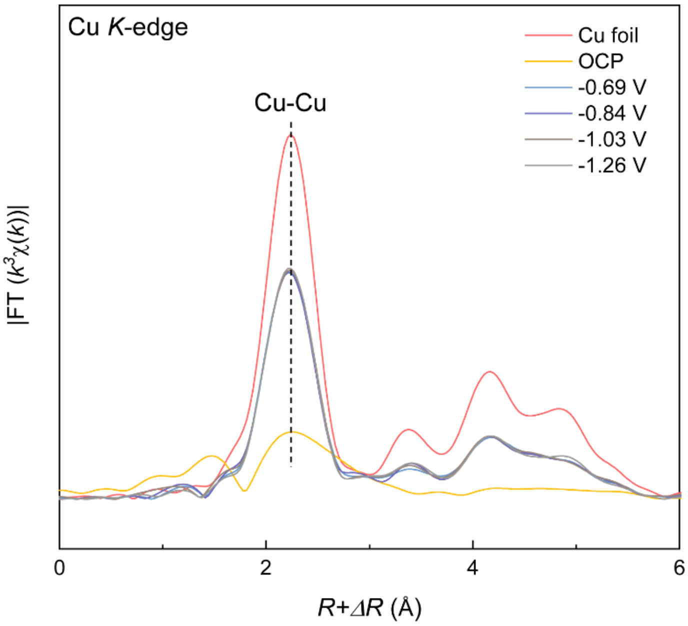

Supplementary Fig. 8 | *Operando* Cu *K*-edge EXAFS of the $Cu_{92}Sb_5Pd_3$ catalyst under applied potentials during the CO_2RR. The Cu foil is shown as a reference. All potentials were calibrated to the RHE scale.

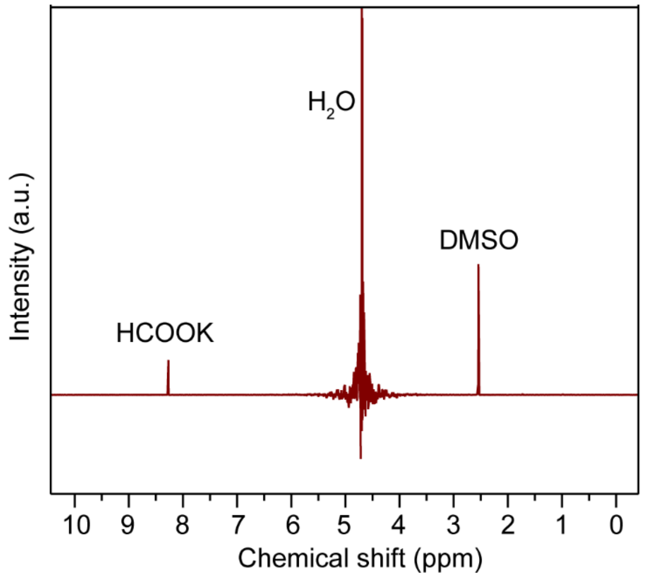

Supplementary Fig. 9 | NMR results of the obtained cathodic electrolyte after the CO_2RR from the $Cu_{92}Sb_5Pd_3$ catalyst. The solution-phase product contained only formate. DMSO was used as an internal standard.

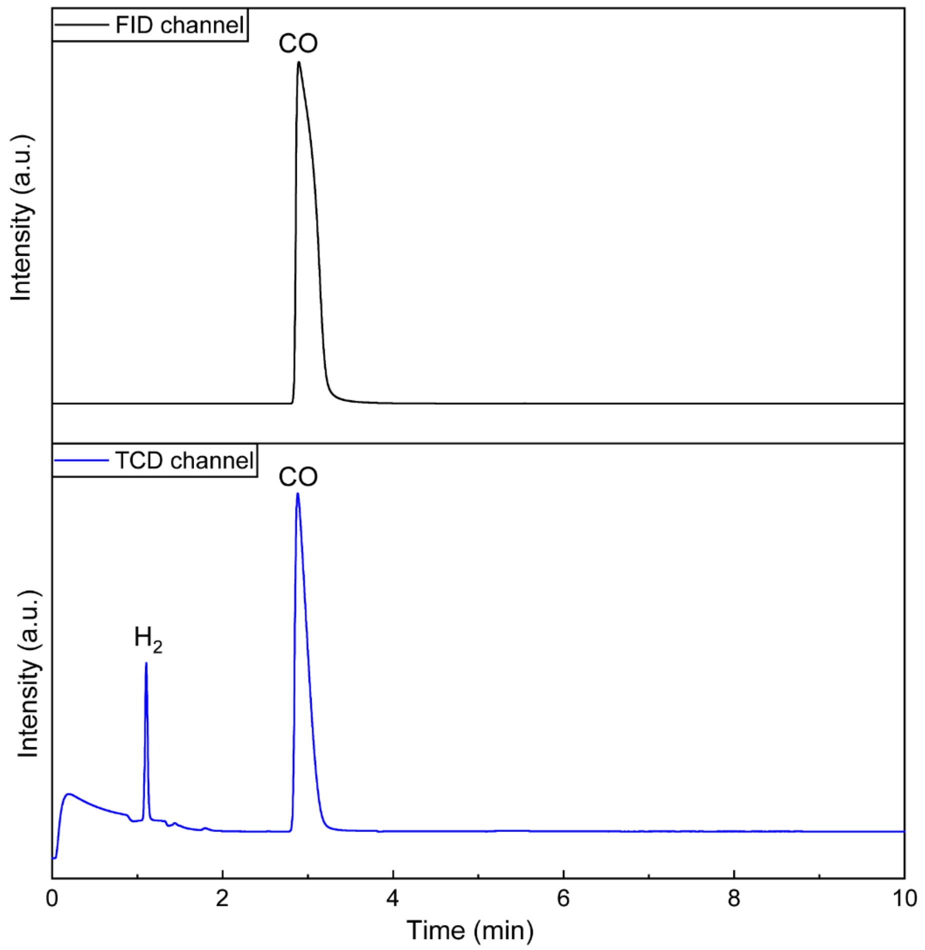

Supplementary Fig. 10 | GC analysis of $Cu_{92}Sb_5Pd_3$ catalyst during CO_2RR. Only CO and H_2 were major gas-phase products.

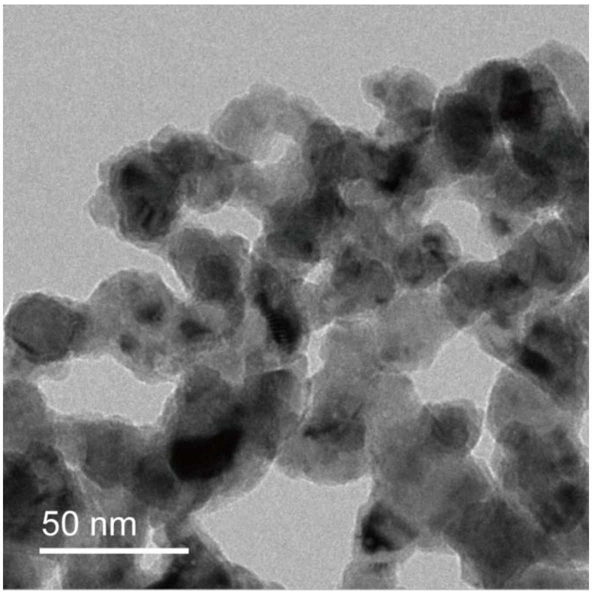

Supplementary Fig. 11 | TEM images of as-synthesized Cu catalyst.

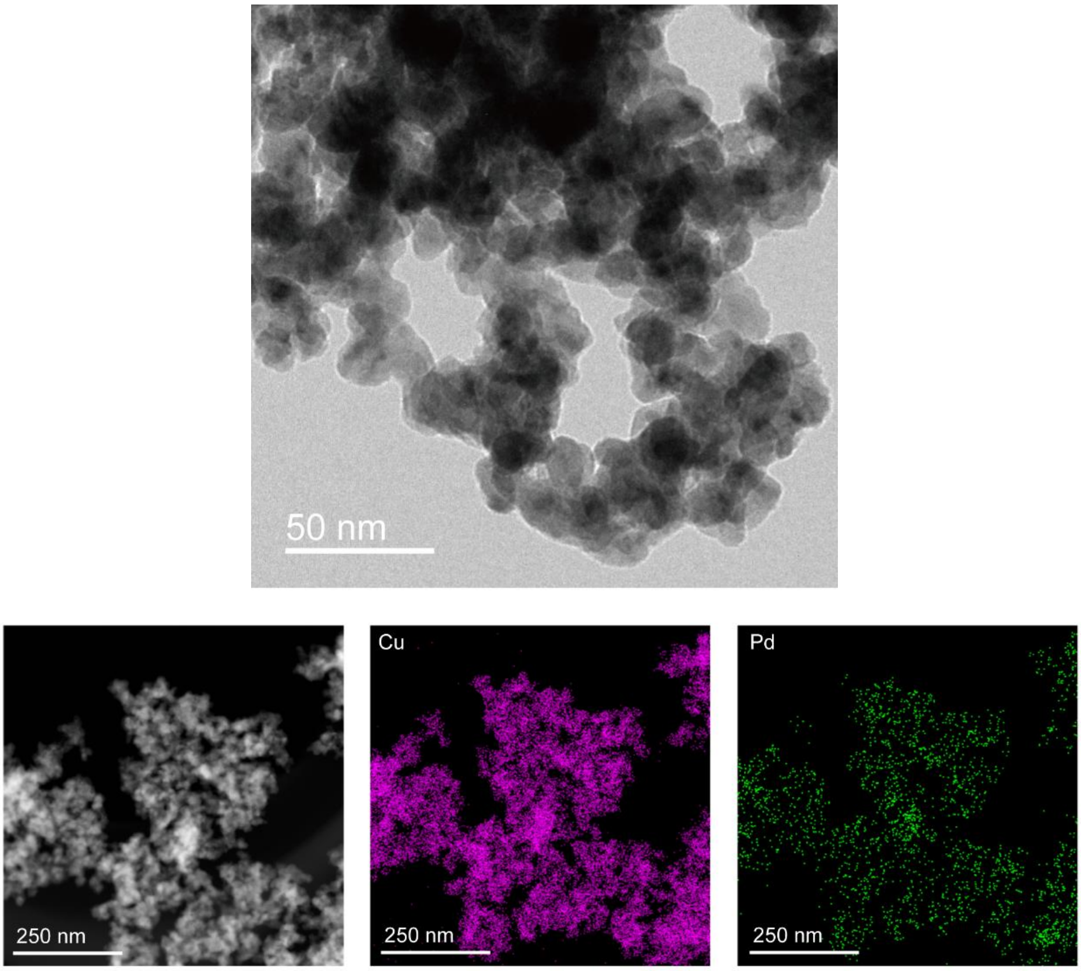

Supplementary Fig. 12 | TEM images and large-scale EDS mapping of the as-synthesized $Cu_{97}Pd_3$ catalyst. No segregation was observed, precluding the existence of Pd particles.

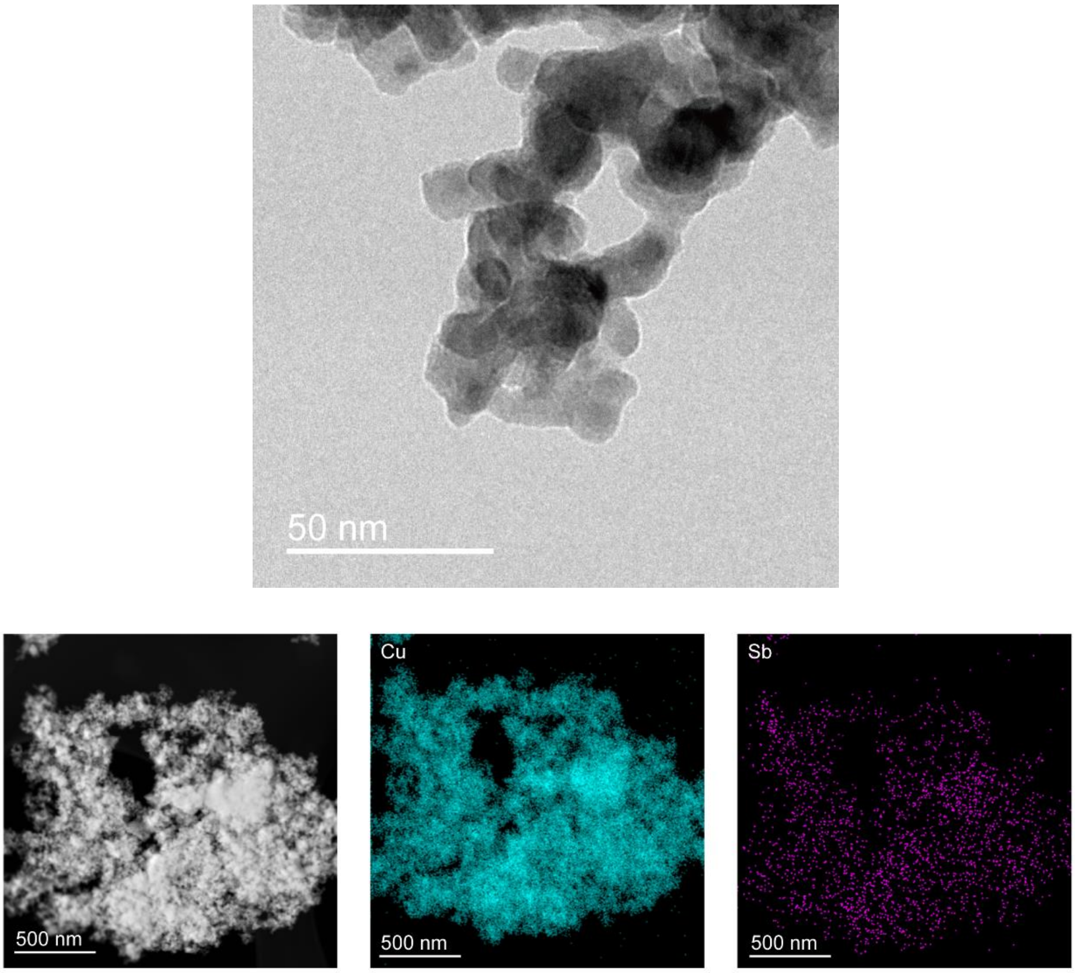

Supplementary Fig. 13 | TEM images and large-scale EDS mapping of the as-synthesized $Cu_{95}Sb_5$ catalyst. No segregation was detected, precluding the presence of Sb particles.

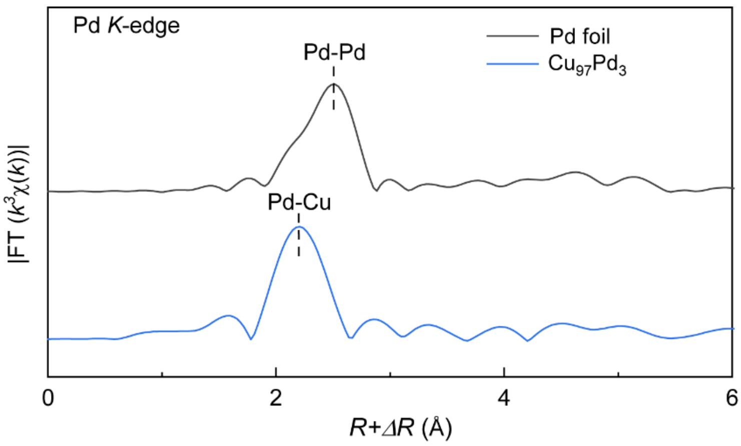

Supplementary Fig. 14 | *Ex situ* EXAFS spectra at the Pd *K*-edge of the $Cu_{97}Pd_3$ catalyst. The spectrum of the Pd foil is shown as a reference. Only Pd-Cu bonds with no signal of Pd-Pd bonds were detected, confirming the presence of atomically dispersed Pd sites in $Cu_{97}Pd_3$.

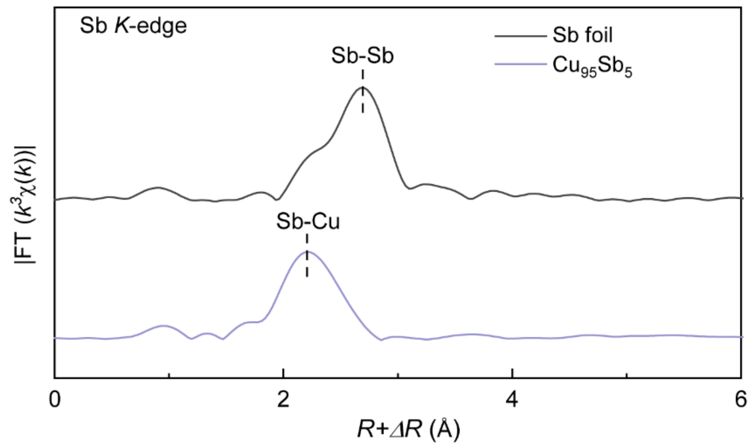

Supplementary Fig. 15 | *Ex situ* EXAFS spectra at the Sb *K*-edge of the $Cu_{95}Sb_5$ catalyst. The spectrum of Sb foil is shown as a reference. Only Sb-Cu bonds with no Sb-Sb bonds were detected, confirming the presence of atomically dispersed Sb sites in $Cu_{95}Sb_5$.

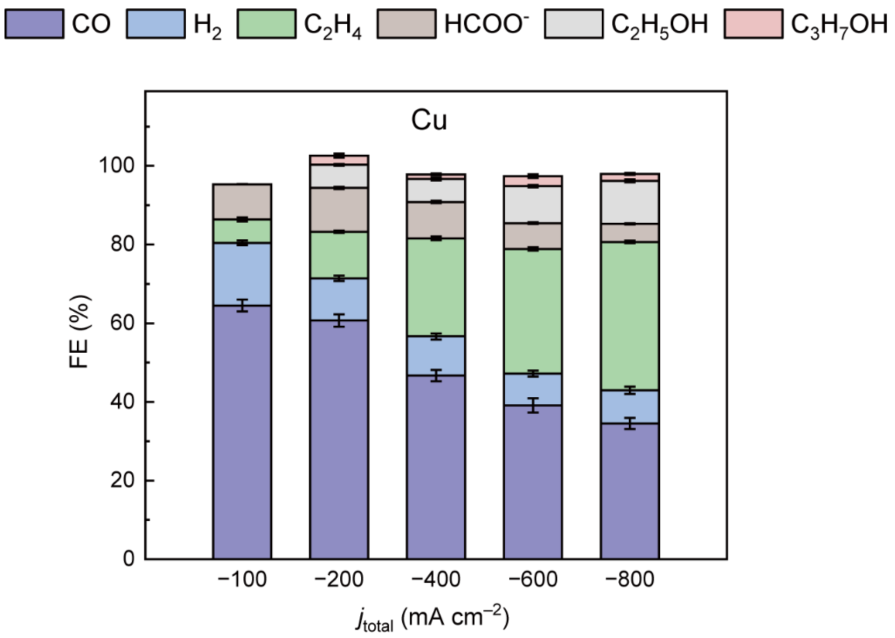

Supplementary Fig. 16 | CO_2RR catalytic performance of pristine Cu as the control sample. From the product analyses, a large amount of C_{2+} products were detected in the case of the Cu sample in comparison with the $Cu_{92}Sb_5Pd_3$ catalyst. This led us to believe that the intrinsic activity and selectivity of Cu were significantly modulated by the co-introduction of the Pd and Sb components. The error bars correspond to the standard deviation of three independent measurements with 0.5 M $KHCO_3$ as the electrolyte.

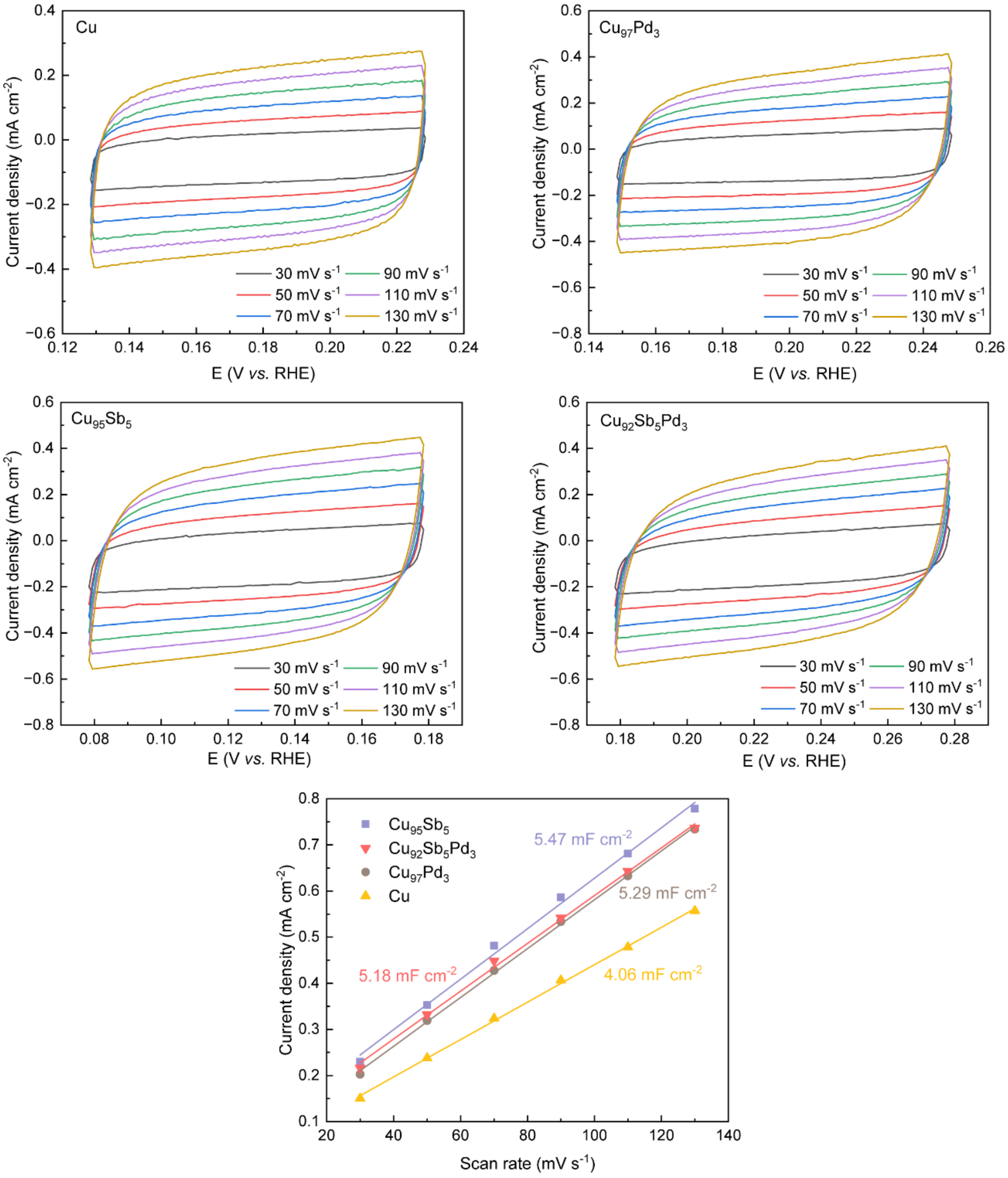

Supplementary Fig. 17 | ECSA results for the four catalysts. Considering that the calculated ECSA results of the four samples were similar, the influence of different ECSAs could be rationally ignored.

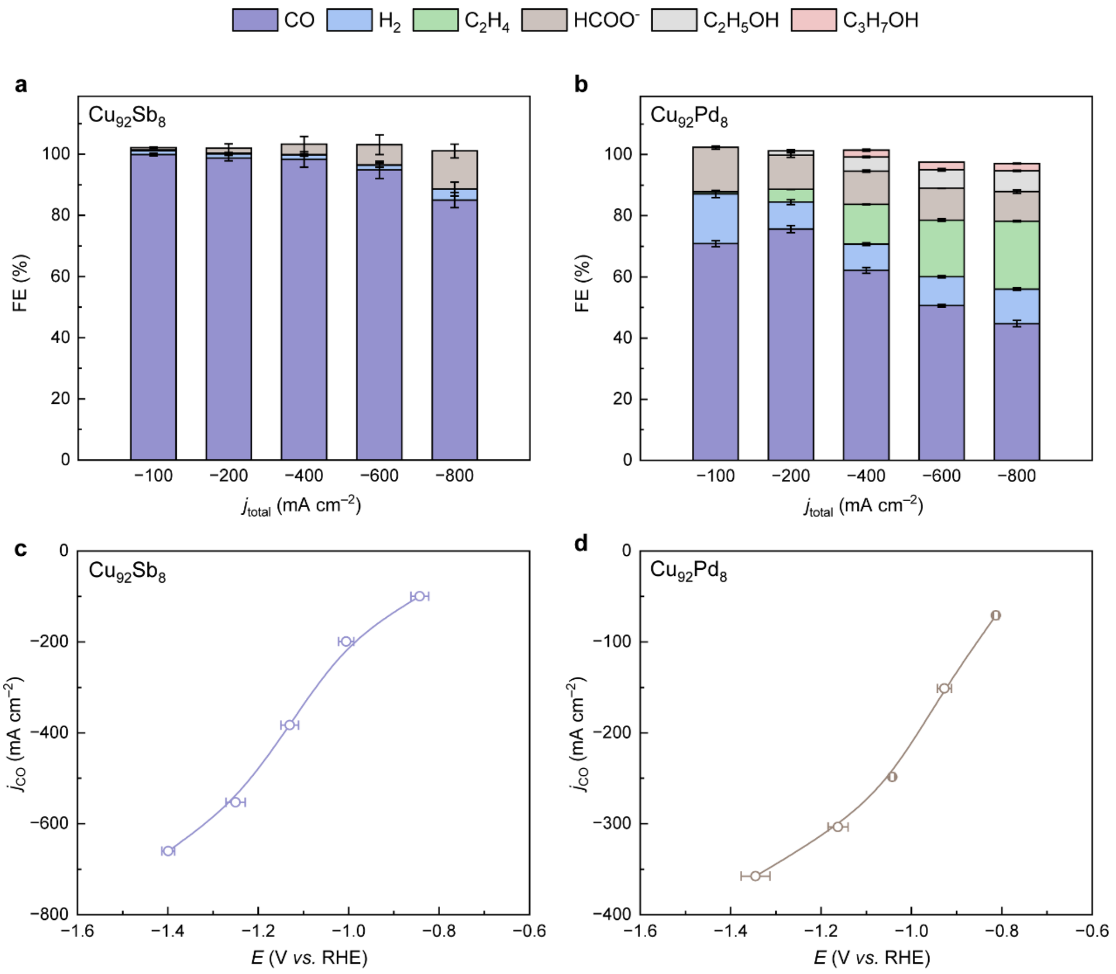

Supplementary Fig. 18 | CO_2RR catalytic performance of the $Cu_{92}Sb_8$ (a and c) and $Cu_{92}Pd_8$ (b and d) catalysts. The results showed that $Cu_{92}Sb_8$ produced a large amount of formate even under modest current densities. In comparison, $Cu_{92}Pd_8$ failed to suppress C-C coupling on the Cu matrix. The error bars correspond to the standard deviation with 0.5 M $KHCO_3$ as the electrolyte.

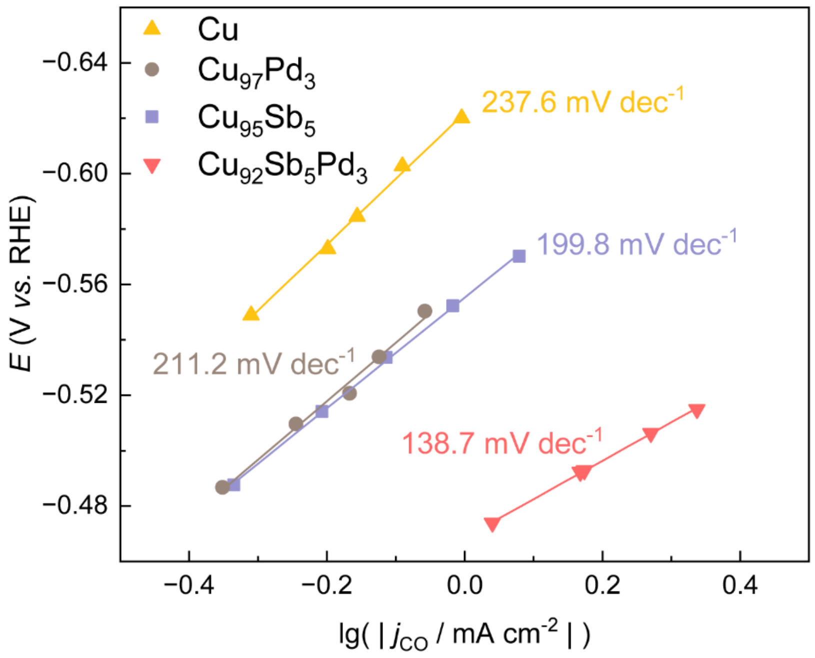

Supplementary Fig. 19 | Tafel plots of the four as-synthesized catalysts. The smaller Tafel slope of $Cu_{92}Sb_5Pd_3$ (138.7 mV dec^{-1}) compared with those of its other three counterparts (Cu as 237.6 mV dec^{-1}, $Cu_{97}Pd_3$ as 211.2 mV dec^{-1} and $Cu_{95}Sb_5$ as 199.8 mV dec^{-1}) indicates an accelerated electron transfer process[13,14]. Note that the deviation from a theoretical value of 118 mV dec^{-1} (**Supplementary Table 4**) was likely due to more complicated electron transfer and electrochemical processes in real reactions[15].

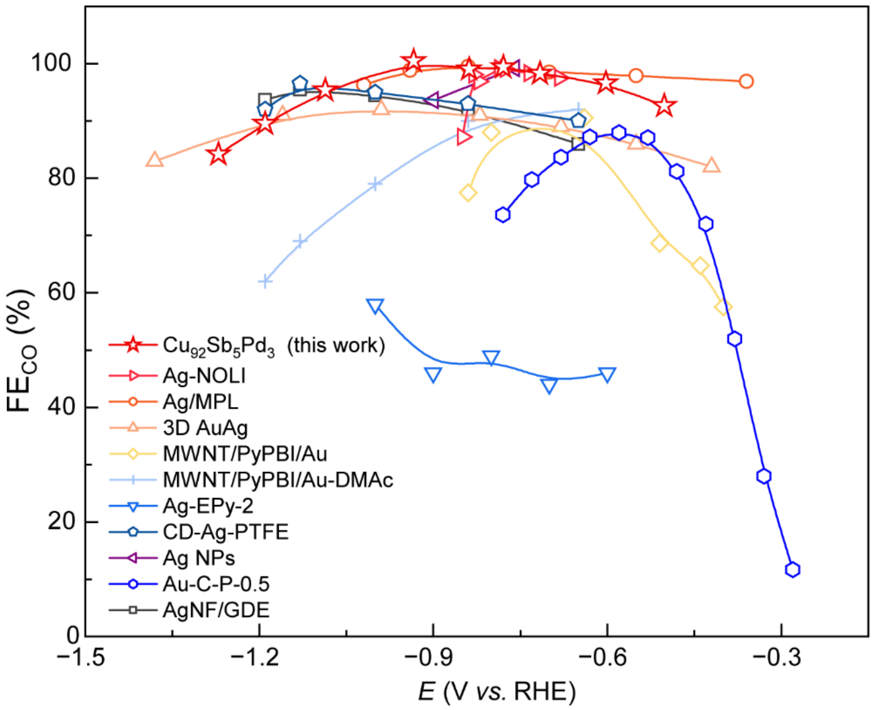

Supplementary Fig. 20 | FE_{CO}-V curves of state-of-the-art noble metal catalysts in flow cell systems during the CO_2RR compared with those of the $Cu_{92}Sb_5Pd_3$ catalyst. The following reference catalysts were used: Ag-NOLI (1 M $KHCO_3$)[3], Ag/MPL (0.1 M $KHCO_3$)[4], 3D AuAg (1 M $KHCO_3$)[5], MWNT/PyPBI/Au (2 M $KHCO_3$)[6], MWNT/PyPBI/Au-DMAc (1 M KCl)[7], Ag-EPy-2 (0.1 M $KHCO_3$)[8], CD-Ag-PTFE (1 M $KHCO_3$)[9], Ag NPs (2 M $KHCO_3$)[10], Au-C-P-0.5 (1 M $KHCO_3$)[11] and AgNF/GDE (1 M KCl)[12]. Strikingly, the $Cu_{92}Sb_5Pd_3$ catalyst maintained exclusive selectivity towards CO over a wide potential range compared with its elaborately designed noble metal counterparts. All potentials were calibrated to the RHE scale.

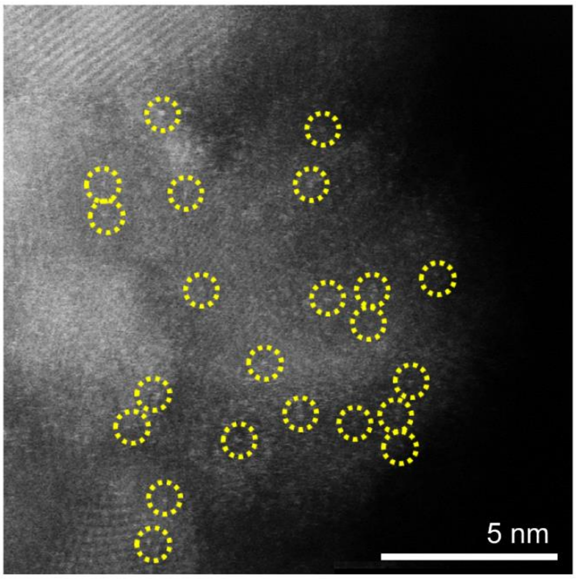

Supplementary Fig. 21 | HAADF-STEM image of $Cu_{92}Sb_5Pd_3$ after the CO_2RR. The yellow circles highlight single Sb/Pd atoms. Sb/Pd aggregation did not occur, demonstrating the stability of the $Cu_{92}Sb_5Pd_3$ catalyst after the CO_2RR.

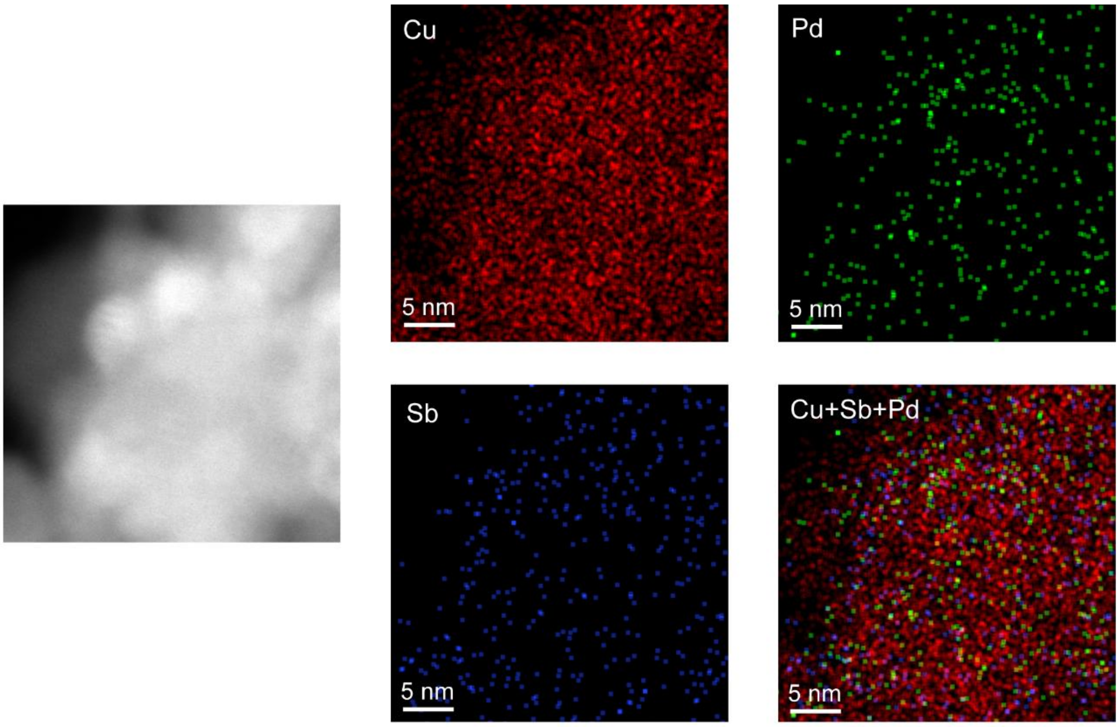

Supplementary Fig. 22 | HAADF-STEM and STEM-EDS mapping of $Cu_{92}Sb_5Pd_3$ after the CO_2RR. STEM-EDS confirmed an even distribution of Sb and Pd in the Cu base without noticeable aggregation in $Cu_{92}Sb_5Pd_3$ after the CO_2RR.

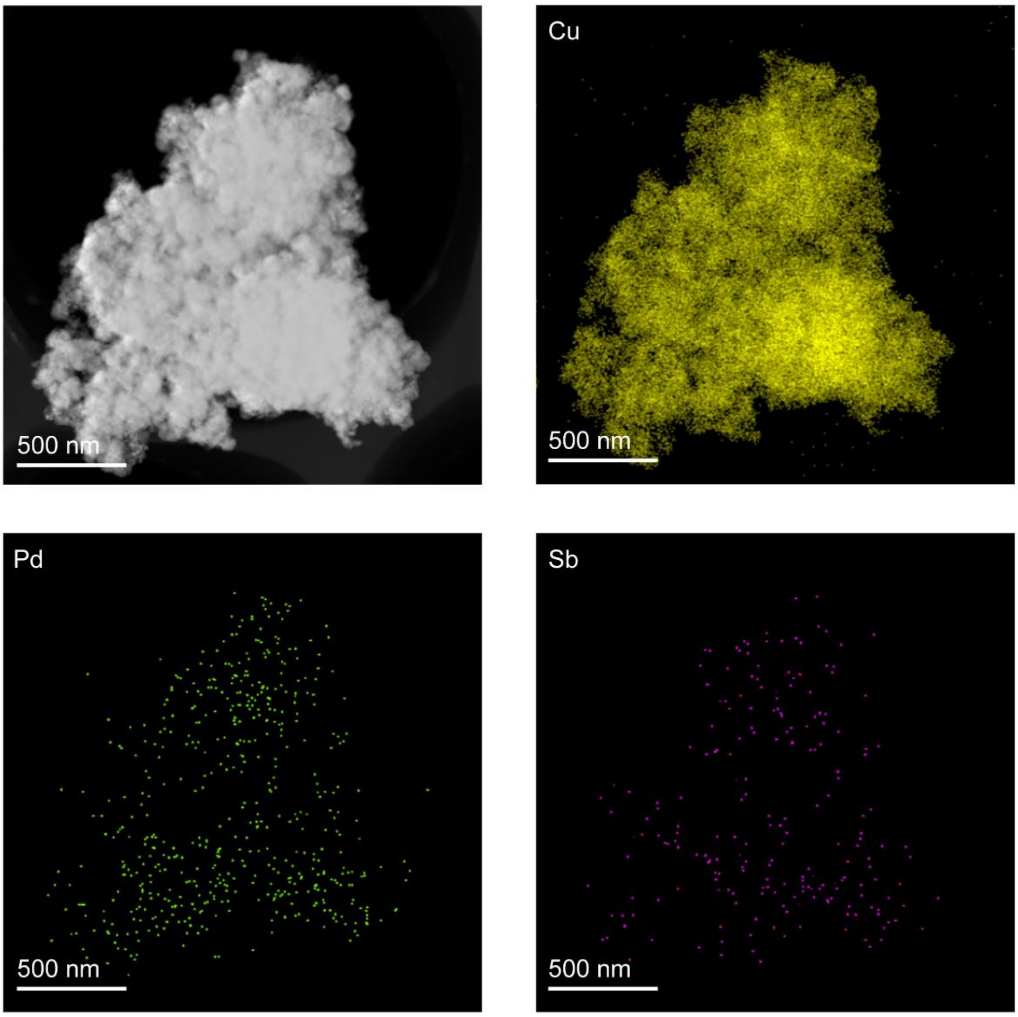

Supplementary Fig. 23 | Large-scale EDS mapping of $Cu_{92}Sb_5Pd_3$ after the CO_2RR. Nevertheless, no large aggregations were detected after the CO_2RR, further proving the exclusive stability of the $Cu_{92}Sb_5Pd_3$ catalyst.

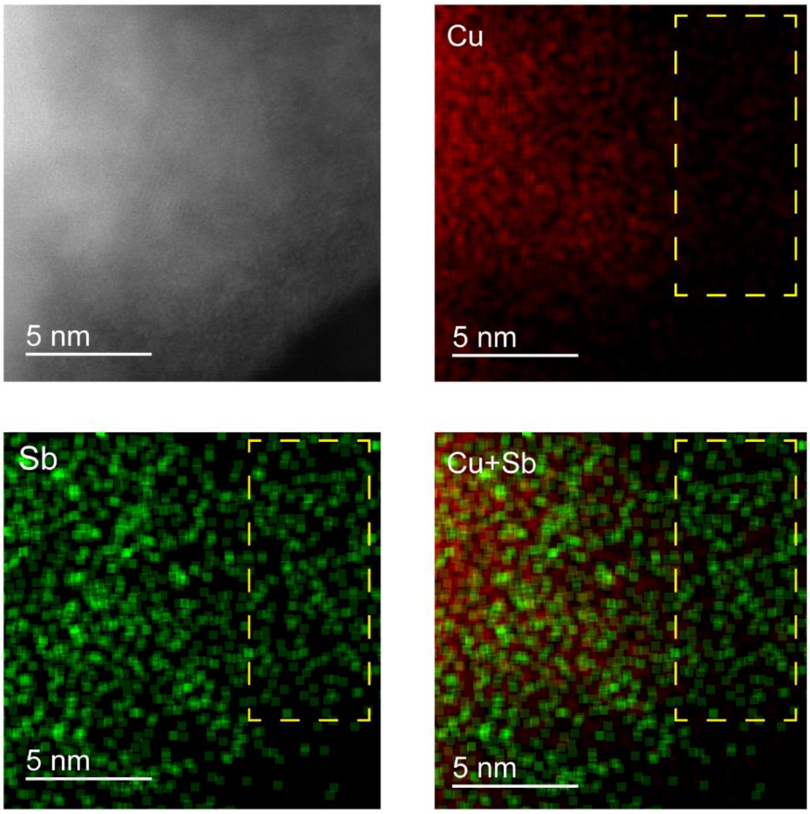

Supplementary Fig. 24 | HAADF-STEM and STEM-EDS mapping of $Cu_{95}Sb_5$ after the CO_2RR. Sb segregation, marked by yellow squares, was identified via STEM-EDS mapping, in which a high concentration of Sb contrasted sharply with a negligible amount of Cu. These results confirmed the inferior stability of $Cu_{95}Sb_5$ after the CO_2RR.

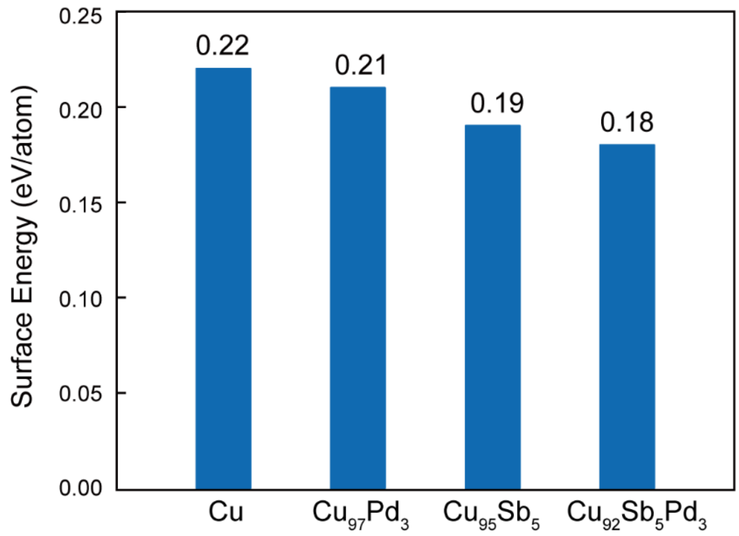

Supplementary Fig. 25 | Surface energies of Cu, $Cu_{97}Pd_3$, $Cu_{95}Sb_5$, and $Cu_{92}Sb_5Pd_3$. This result confirmed the improved stability of the $Cu_{92}Sb_5Pd_3$ single-atom alloy catalyst by codoping Sb and Pd on Cu.

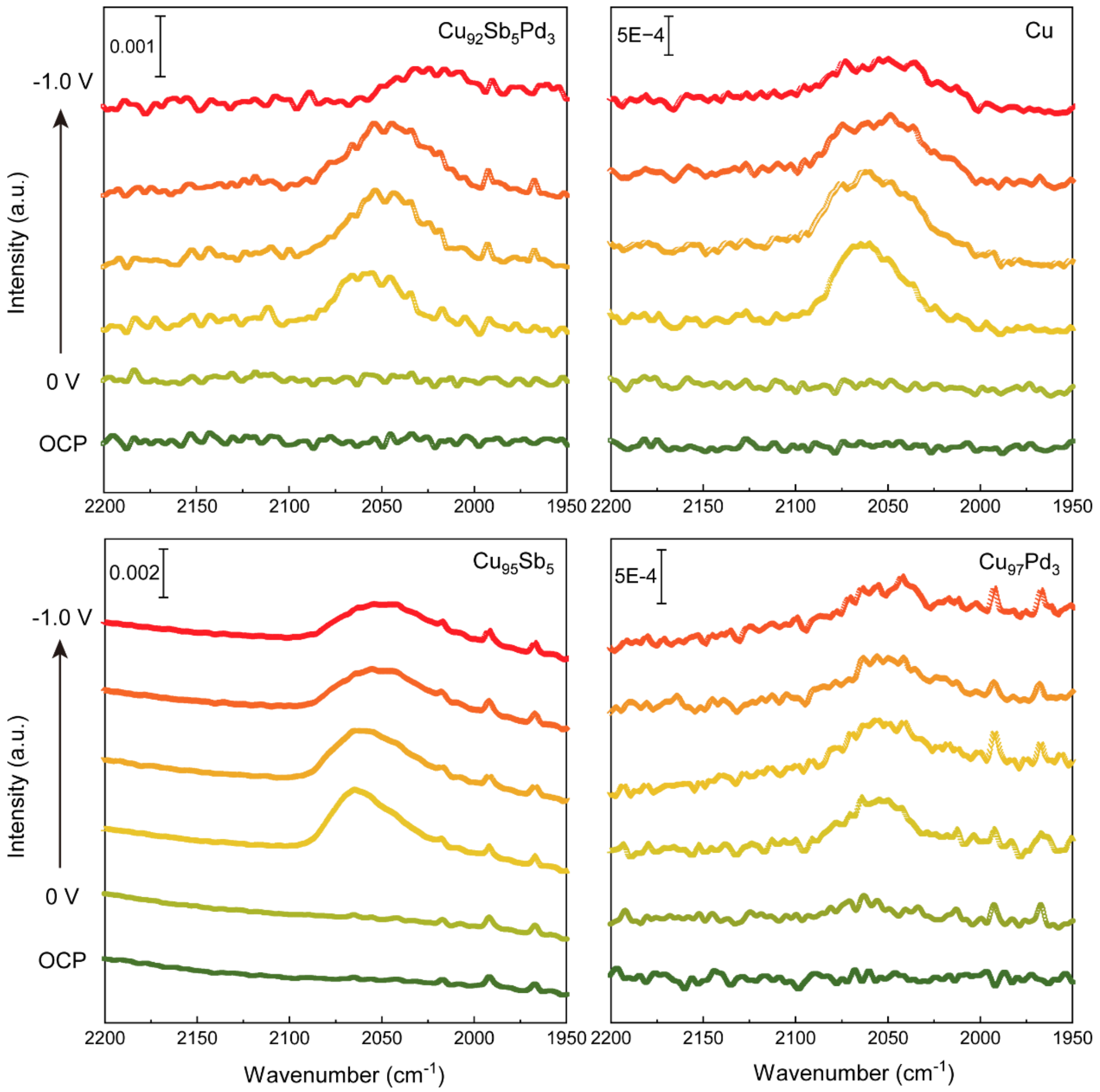

Supplementary Fig. 26 | *In situ* ATR-SEIRAS spectra of four samples during the CO_2RR. Note that when increasing the bias to -1.0 V *vs.* RHE, the CO* peak vanished on $Cu_{92}Sb_5Pd_3$ but was still present on other samples, indicating easier desorption of CO* intermediates from the $Cu_{92}Sb_5Pd_3$ catalyst surface to form gaseous CO. All potentials were calibrated to the RHE scale.

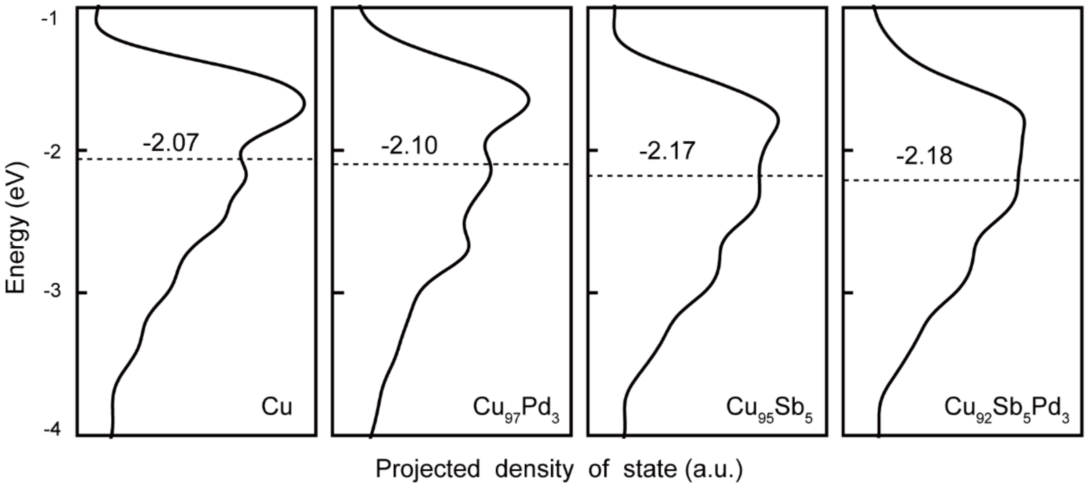

Supplementary Fig. 27 | DFT-calculated projected *d*-band states for Cu sites in different samples. The calculation was based on the structures provided in **Supplementary Fig. S28**. The trend of the *d*-band centers was in accordance with the SVBS measurements. Note that the calculated projected *d*-band states were only on Cu sites; therefore, their deviation from the SVBS measurements was reasonable.

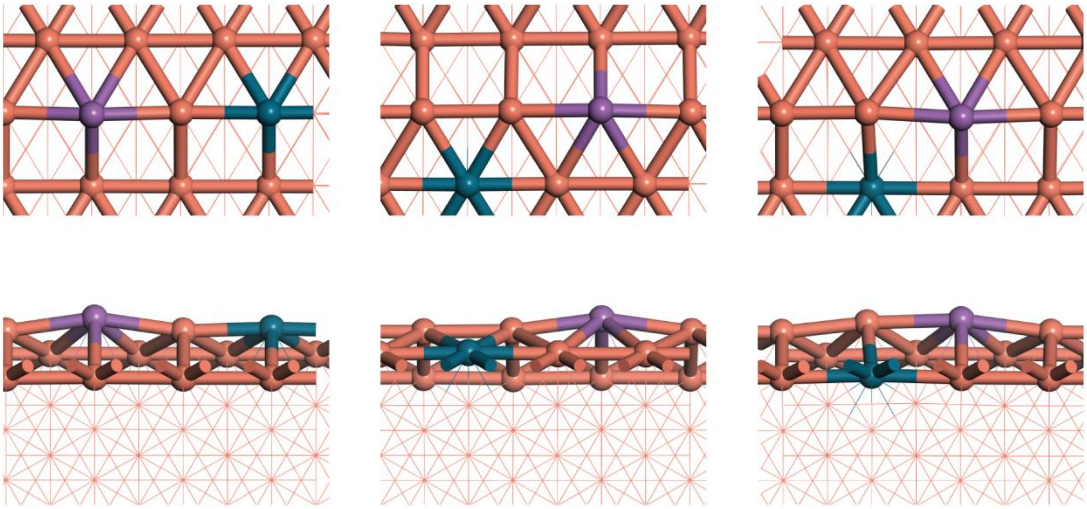

$Cu_{92}Sb_5Pd_3$ (211) $Cu_{92}Sb_5Pd_3$ (211)-1 $Cu_{92}Sb_5Pd_3$ (211)-2

Supplementary Fig. 28 | The top and side view of the theoretical structures.

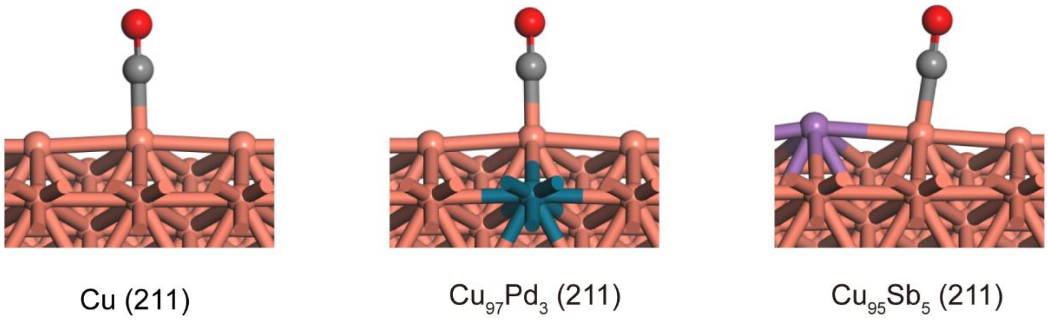

Supplementary Fig. 29 | The adsorption states of CO* on Cu (211), $Cu_{97}Pd_3$ (211) and $Cu_{95}Sb_5$ (211).

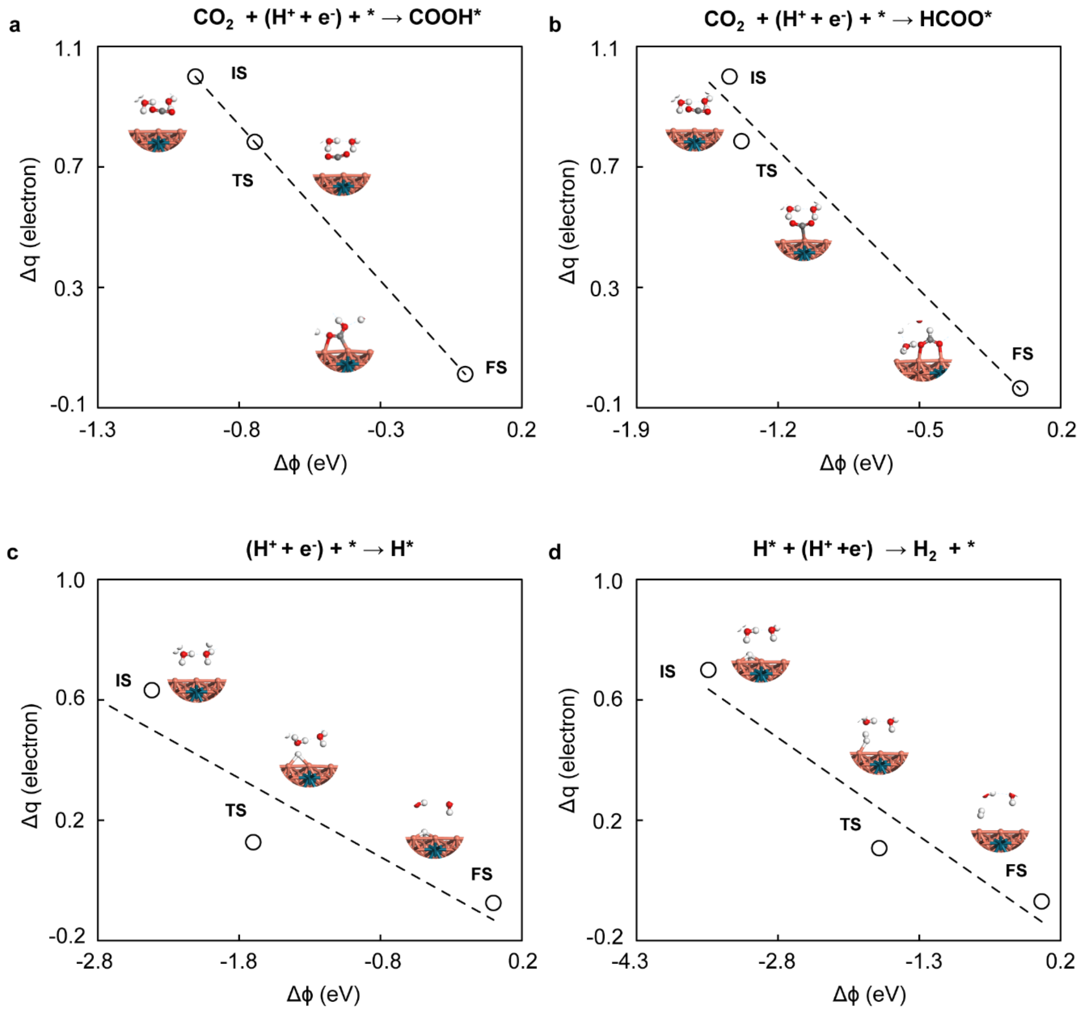

Supplementary Fig. 30 | Calculated charge transfer (Δ*q*) and *Φ* on electrochemical interface at the initial state (IS), transition state (TS), and final state (FS) for COOH* formation (a), HCOO* formation (b), Volmer (c) and Heyrovsky (d) steps over $Cu_{97}Pd_3$ (211).

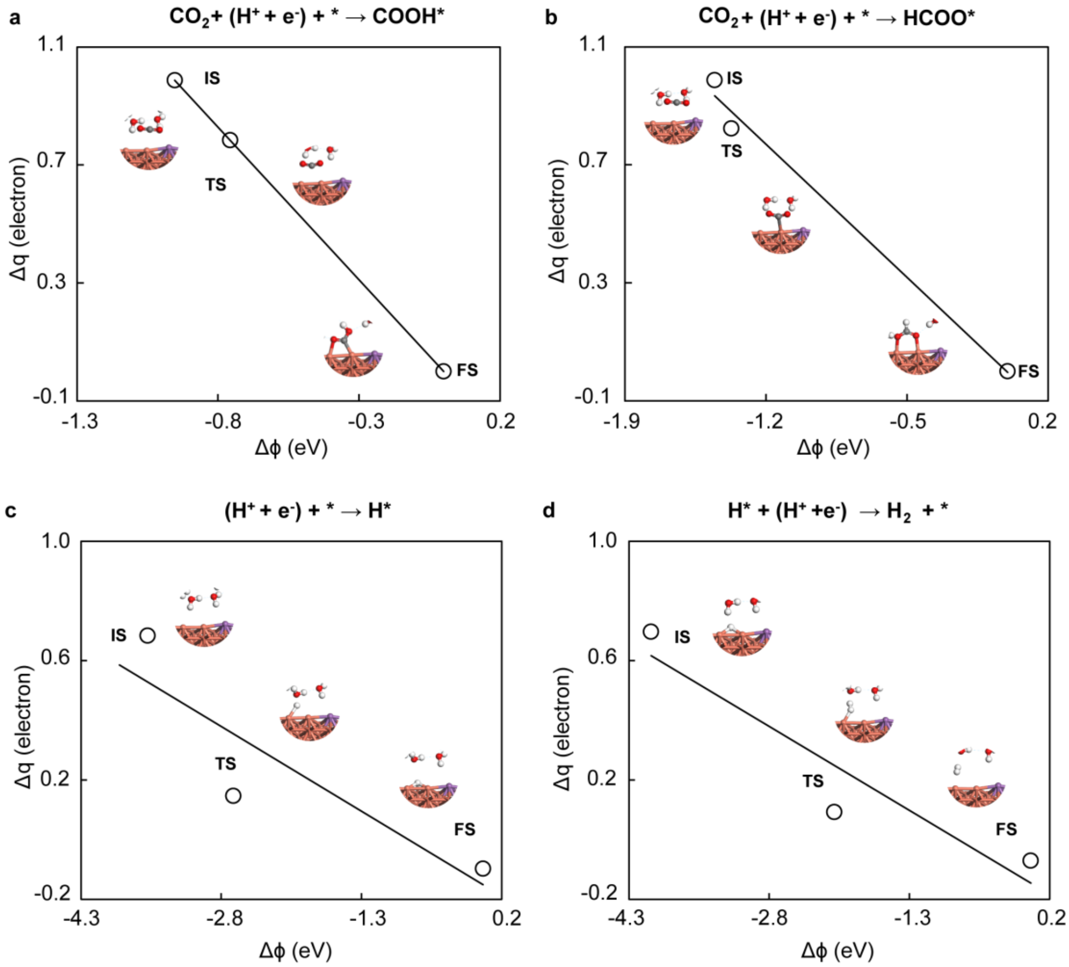

Supplementary Fig. 31 │ Calculated charge transfer (Δ*q*) and *Φ* on electrochemical interface at the initial state (IS), transition state (TS), and final state (FS) for COOH* formation (a), HCOO* formation (b), Volmer (c) and Heyrovsky (d) steps over $Cu_{95}Sb_5$ (211).

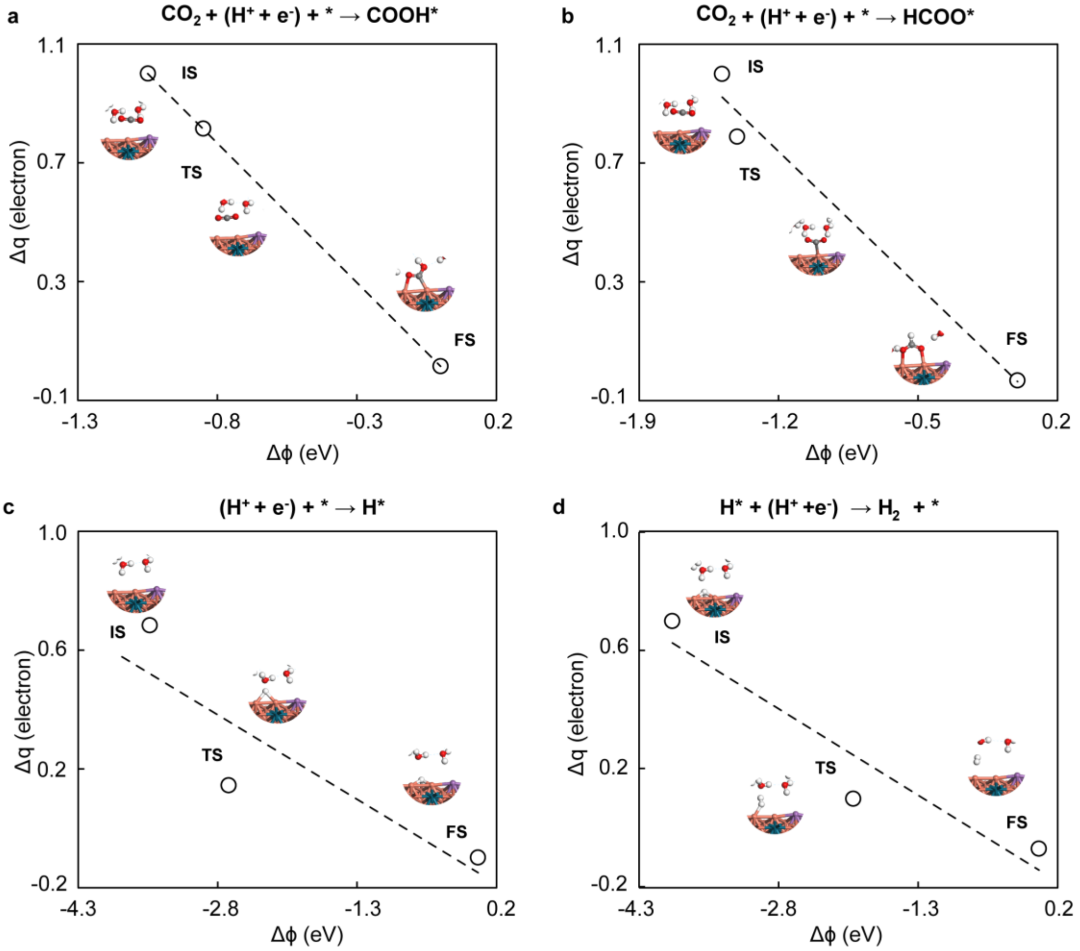

Supplementary Fig. 32 | Calculated charge transfer (Δ*q*) and *Φ* on electrochemical interface at the initial state (IS), transition state (TS), and final state (FS) for COOH* formation (a), HCOO* formation (b), Volmer (c) and Heyrovsky (d) steps over $Cu_{92}Sb_5Pd_3$ (211)-2.

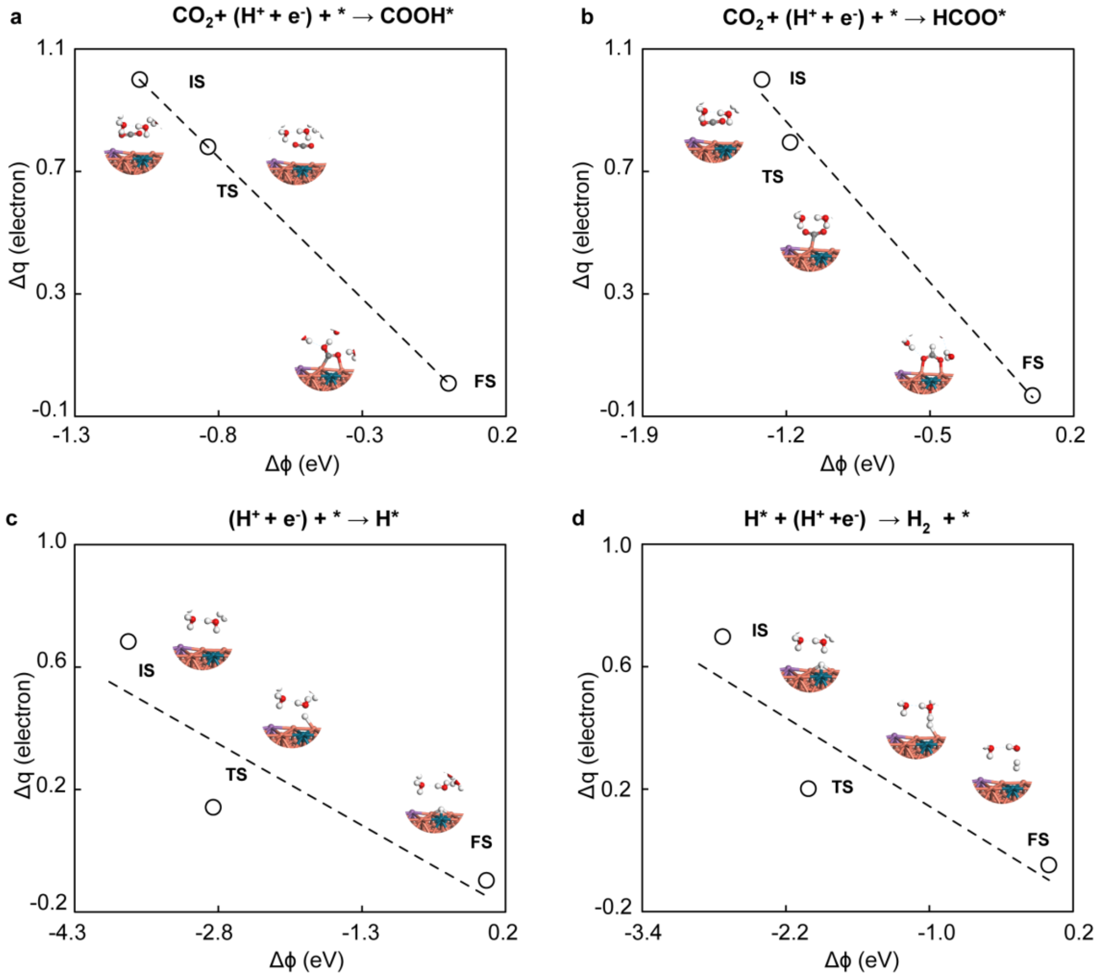

Supplementary Fig. 33 | Calculated charge transfer (Δ*q*) and *Φ* on electrochemical interface at the initial state (IS), transition state (TS), and final state (FS) for COOH* formation (a), HCOO* formation (b), Volmer (c) and Heyrovsky (d) steps over $Cu_{92}Sb_5Pd_3$ (211)-1.

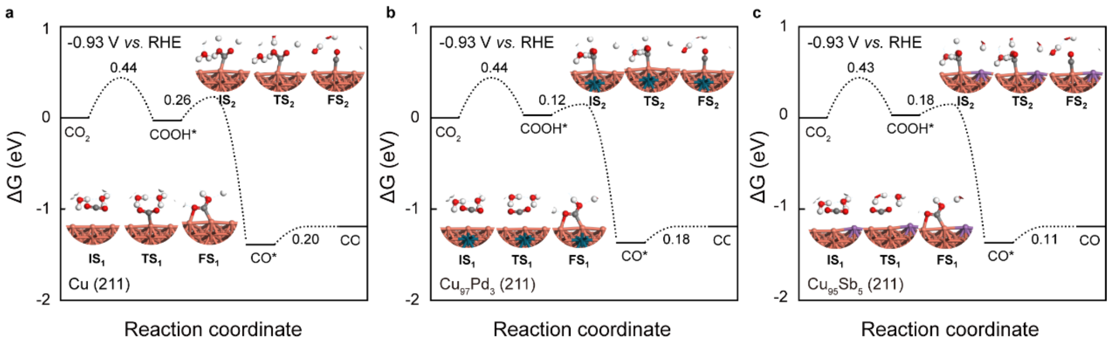

Supplementary Fig. 34 | CO_2RR to CO on Cu (211) (a), $Cu_{97}Pd_3$ (211) (b) and $Cu_{95}Sb_5$ (211) (c). The initial (IS), transition (TS), and final (FS) structures are shown as insets, where Cu, Sb, Pd, C, O, and H are represented in orange, purple, green, gray, red, and white, respectively.

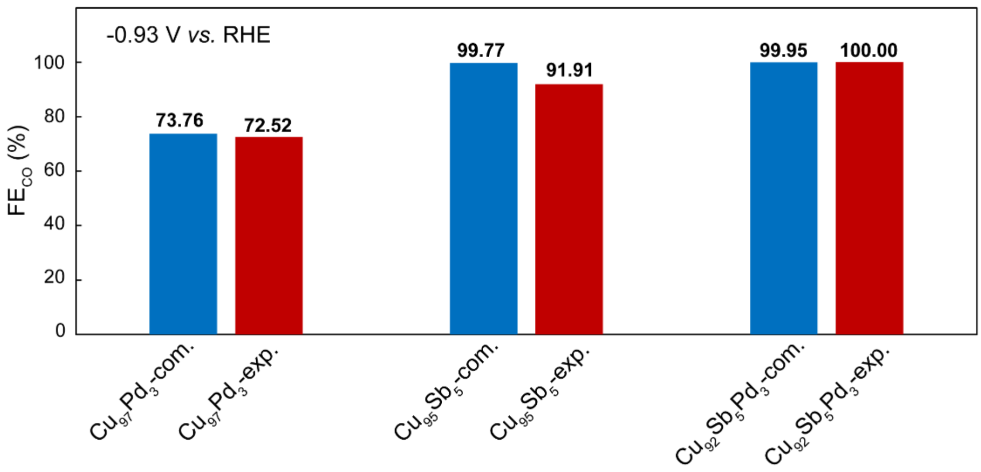

Supplementary Fig. 35 | Comparison between the calculated FE$_{CO}$ from microkinetic simulations on $Cu_{97}Pd_3$ (211), $Cu_{95}Sb_5$ (211) and $Cu_{92}Sb_5Pd_3$ (211)-2 and the experimental FE$_{CO}$ on $Cu_{97}Pd_3$, $Cu_{95}Sb_5$ and $Cu_{92}Sb_5Pd_3$.

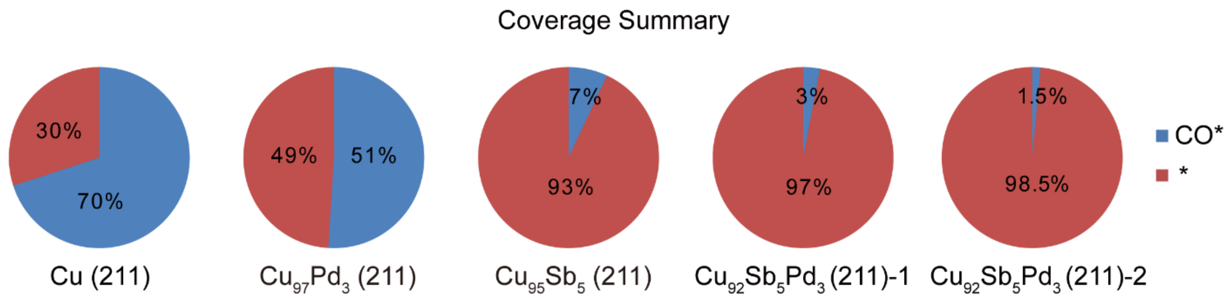

Supplementary Fig. 36 | Coverage summary at the steady state for the CO_2RR on different models at -0.93 V *vs.* RHE. The symbol * refers to the free sites on the surface.

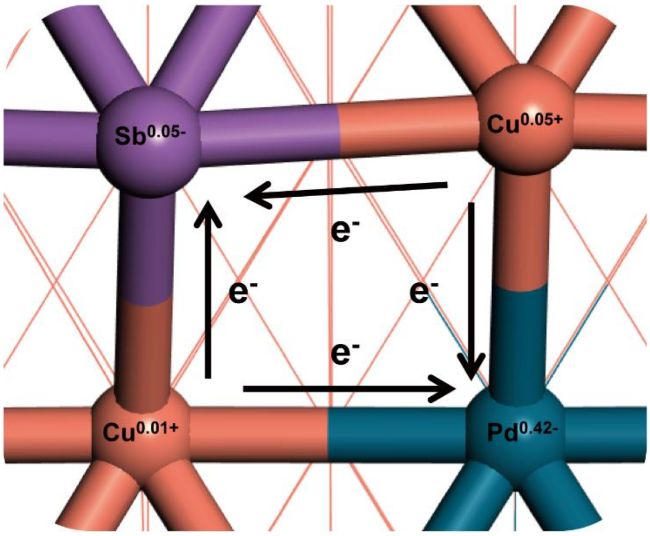

Supplementary Fig. 37 | Bader charge analysis of $Cu_{92}Sb_5Pd_3$. Copper atoms present partial electron-deficient states in $Cu_{92}Sb_5Pd_3$, which confirms the charge redistribution between Sb/Pd additions and the Cu matrix, consistent with former *operando* XAS results.

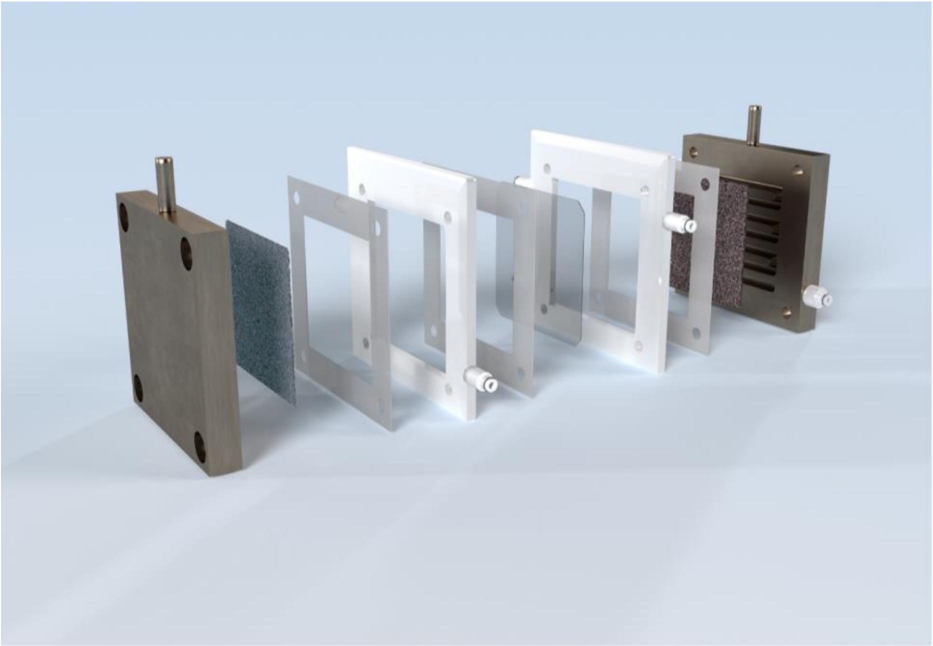

Supplementary Fig. 38 | Schematic illustration of the flow-cell configuration.

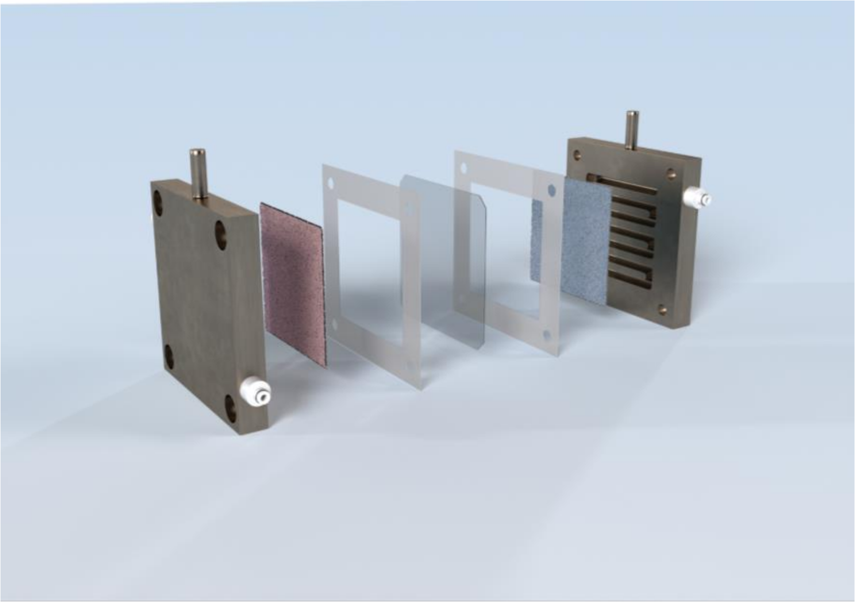

Supplementary Fig. 39 | Schematic illustration of the MEA used in the stability test.

Supplementary Table 1. EXAFS fitting parameters at the Sb *K*-edge for the $Cu_{92}Sb_5Pd_3$ sample (S_0^2 = 0.803).

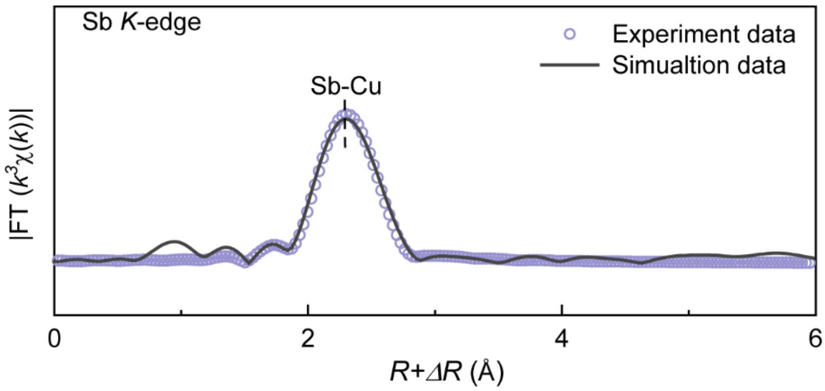

Sample	Shell	N^a	R(Å)^b	σ^2(Å^2)^c	ΔE_0 (eV)^d	*R* factor
**Sb foil**	Sb-Sb	3.0	2.92	0.0050	8.2	0.0003
**$Cu_{92}Sb_5Pd_3$**	Sb-Cu	3.2	2.69	0.0060	2.1	0.0010

$^a N$: coordination number; $^b R$: bond distance; $^c \sigma^2$: Debye-Waller factor; ^{d} ΔE_0: inner potential correction. *R* factor: goodness of fit. S_0^2 was set to 0.803, according to the experimental EXAFS fit of the Sb foil reference by fixing coordination numbers as the known crystallographic value.

Supplementary Table 2. EXAFS fitting parameters at the Pd *K*-edge for the $Cu_{92}Sb_5Pd_3$ sample (S_0^2 = 0.830).

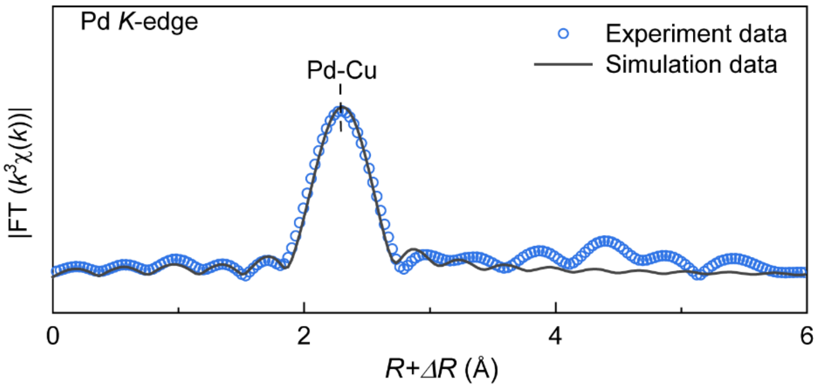

Sample	Shell	N^a	R(Å)^b	σ^2(Å^2)^c	ΔE_0 (eV)^d	*R* factor
**Pd foil**	Pd-Pd	12.0	2.74	0.0054	1.6	0.0007
**$Cu_{92}Sb_5Pd_3$**	Pd-Cu	2.4	2.59	0.0019	11.6	0.0095

$^a N$: coordination number; $^b R$: bond distance; $^c\sigma^2$: Debye-Waller factor; ^{d} ΔE_0: inner potential correction. *R* factor: goodness of fit. S_0^2 was set to 0.830, according to the experimental EXAFS fit of the Pd foil reference by fixing coordination numbers as the known crystallographic value.

Supplementary Table 3. ICP-AES results for different control samples.

	Cu content (at%)	Pd content (at%)	Sb content (at%)
**$Cu_{95}Sb_5$**	94.2	0	5.7
**$Cu_{97}Pd_3$**	97.2	2.8	0
**$Cu_{92}Sb_8$**	92.3	0	7.7
**$Cu_{92}Pd_8$**	92.2	7.8	0

Supplementary Table 4. Tafel slopes for different possible RDSs during CO_2-to-CO conversion (assuming α = 0.5).

**Possible RDSs**	**Type**	**Tafel slope (mV dec^{-1})**
$CO_2 + * + e^- \rightarrow *CO_2^-$	Electron transfer	118
$*CO_2^- + H_2O \rightarrow *COOH + OH^-$	Proton transfer	59
$*COOH + e^- \rightarrow *COOH^-$	Electron transfer	39
$*COOH^- \rightarrow *CO + OH^-$	Proton transfer	30
$*CO \rightarrow CO + *$	Desorption	30

Supplementary Table 5. Performances of reported state-of-the-art noble metal catalysts in stability tests.

Cathode	Catholyte	Cell structure	Current density (mA cm^{-2})	FE_{CO} (%)	Stability (h)	Ref.
$Cu_{92}Sb_5Pd_3$	None	MEA	-100	>95	528	This work
Au/C-P-0.5	1 M KOH	Flow cell	-100	90	10	11
Ag GDE	1 M KOH	Flow cell	-41.5	90	50	16
Ag	7 M KOH	Flow cell	~ -90	>85	10	17
Ag	None	Flow cell	-100	65	24	18
Ag NF	0.5 M $KHCO_3$	Home-made electrolyzer	-13	95	10	12
			-24	90	10	
			-40	>77	8	

Supplementary Table 6. Calculated barriers (eV) of the considered elementary steps for the CO_2RR and HER on different surfaces at -0.93V vs. RHE.

Reaction	$Cu_{92}Sb_5Pd_3$ (211)-1	$Cu_{92}Sb_5Pd_3$ (211)-2	$Cu_{97}Pd_3$ (211)	$Cu_{95}Sb_5$ (211)
$CO_2 + (H^+ + e^-) + * \rightarrow COOH^*$	0.43	0.41	0.44	0.43
$COOH^* + (H^+ + e^-) \rightarrow CO^* + H_2O$	0.12	0.11	0.12	0.18
$CO^* \rightarrow CO + *$	0.09	0.07	0.18	0.11
$CO_2 + (H^+ + e^-) + * \rightarrow HCOO^*$	0.54	0.62	0.51	0.59
$HCOO^* + (H^+ + e^-) \rightarrow HCOOH + *$	0.00	0.00	0.00	0.00
$(H^+ + e^-) + * \rightarrow H^*$	0.75	0.63	0.59	0.73
$H^* + (H^+ + e^-) \rightarrow H_2 + *$	0.25	0.24	0.13	0.23

Supplementary Table 7. Calculated reaction free energies (eV) of the considered elementary steps for the CO_2RR and HER on different surfaces at -0.93V vs. RHE.

Reaction	$Cu_{92}Sb_5Pd_3$ (211)-1	$Cu_{92}Sb_5Pd_3$ (211)-2	$Cu_{97}Pd_3$ (211)	$Cu_{95}Sb_5$ (211)
$CO_2 + (H^+ + e^-) + * \rightarrow COOH*$	0.10	0.11	0.03	0.05
$COOH* + (H^+ + e^-) \rightarrow CO* + H_2O$	-1.38	-1.37	-1.40	-1.36
$CO* \rightarrow CO + *$	0.09	0.07	0.18	0.11
$CO_2 + (H^+ + e^-) + * \rightarrow HCOO*$	-0.66	-0.68	-0.81	-0.73
$HCOO* + (H^+ + e^-) \rightarrow HCOOH + *$	-0.40	-0.39	-0.25	-0.34
$(H^+ + e^-) + * \rightarrow H*$	-0.86	-0.83	-0.83	-0.82
$H* + (H^+ + e^-) \rightarrow H_2 + *$	-1.00	-1.03	-1.03	-1.04

Supplementary Table 8. TOFs of different products for the CO_2RR and HER on different surfaces at -0.93 V *vs.* RHE.

	CO (s^{-1})	C_{2+} (s^{-1})	HCOOH (s^{-1})	H_2 (s^{-1})
$Cu_{92}Sb_5Pd_3$ (211)-1	3.62×10^5	3.79×10^{-4}	5.30×10^3	1.57×10^0
$Cu_{92}Sb_5Pd_3$ (211)-2	7.97×10^5	9.22×10^{-9}	2.40×10^2	1.63×10^2
$Cu_{97}Pd_3$ (211)	1.23×10^5	5.25×10^3	1.15×10^4	7.66×10^2
$Cu_{95}Sb_5$ (211)	3.49×10^5	2.74×10^0	7.66×10^2	3.41×10^0

Supplementary References

1. Manzoor, A., Pandey, S., Chakraborty, D. *et al.* Entropy contributions to phase stability in binary random solid solutions. *Npj. Comput. Mater.* **4**, 47 (2018).
2. Xin, Y. *et al.* High-Entropy Alloys as a Platform for Catalysis: Progress, Challenges, and Opportunities. *ACS Catal.* **10 (19)**, 11280-11306 (2020).
3. Kim, D. *et al.* Selective CO_2 electrocatalysis at the pseudocapacitive nanoparticle/ordered-ligand interlayer. *Nat. Energy* **5**, 1032-1042 (2020).
4. Wang, R. *et al.* Maximizing Ag Utilization in High-Rate CO_2 Electrochemical Reduction with a Coordination Polymer-Mediated Gas Diffusion Electrode. *ACS Energy Lett.* **4**, 2024-2031 (2019).
5. Ozden, A. *et al.* Gold Adparticles on Silver Combine Low Overpotential and High Selectivity in Electrochemical CO_2 Conversion. *ACS Appl. Energy Mater.* **4**, 7504-7512 (2021).
6. Verma, S. *et al.* Insights into the Low Overpotential Electroreduction of CO_2 to CO on a Supported Gold Catalyst in an Alkaline Flow Electrolyzer. *ACS Energy Lett.* **3**, 193-198 (2017).
7. Jhong, H. M. *et al.* Gold Nanoparticles on Polymer-Wrapped Carbon Nanotubes: An Efficient and Selective Catalyst for the Electroreduction of CO_2. *Chemphyschem* **18**, 3274-3279 (2017).
8. Abdinejad, M. *et al.* CO_2 Electrolysis via Surface-Engineering Electrografted Pyridines on Silver Catalysts. *ACS Catal.* **12**, 7862-7876 (2022).
9. Dinh, C.-T., García de Arquer, F. P., Sinton, D. & Sargent, E. H. High Rate, Selective, and Stable Electroreduction of CO_2 to CO in Basic and Neutral Media. *ACS Energy Lett.* **3**, 2835-2840 (2018).
10. Verma, S., Lu, X., Ma, S., Masel, R. I. & Kenis, P. J. The effect of electrolyte composition on the electroreduction of CO_2 to CO on Ag based gas diffusion electrodes. *Phys. Chem. Chem. Phys.* **18**, 7075-7084 (2016).
11. Shi, R. *et al.* Efficient wettability-controlled electroreduction of CO_2 to CO at Au/C interfaces. *Nat. Commun.* **11**, 3028 (2020).
12. Wei, L. *et al.* Thiocyanate-Modified Silver Nanofoam for Efficient CO_2 Reduction to CO. *ACS Catal.* **10**, 1444-1453 (2020).
13. Vijay, S. *et al.* Unified mechanistic understanding of CO_2 reduction to CO on transition metal and single atom catalysts. *Nat. Catal.* **4**, 1024-1031 (2021).
14. Lu, Q. *et al.* A selective and efficient electrocatalyst for carbon dioxide reduction. *Nat. Commun.* **5**, 3242 (2014).
15. Fletcher, S. Tafel slopes from first principles. *J. Solid State Electrochem.* **13**, 537-549 (2009).
16. Ye, K., Liu, T., Song, Y., Wang, Q. & Wang, G. Tailoring the interactions of heterogeneous Ag_2S/Ag interface for efficient CO_2 electroreduction. *Appl. Catal. B: Environ.* **296** (2021).
17. Gabardo, C. M. *et al.* Combined high alkalinity and pressurization enable efficient CO_2 electroreduction to CO. *Energy Environ. Sci* **11**, 2531-2539 (2018).
18. Salvatore, D. A. *et al.* Electrolysis of Gaseous CO_2 to CO in a Flow Cell with a Bipolar Membrane. *ACS Energy Lett.* **3**, 149-154 (2018).
